# Occurrence and Dietary Exposure of PFAS in Singapore: Insights from a Total Diet Study

**DOI:** 10.3390/foods14234165

**Published:** 2025-12-04

**Authors:** Ignatius Lim, Ping Shen, Wei Min Ang, Yee Soon Chin, Raymond Rong Sheng Shi, Wesley Zongrong Yu, Sheot Harn Chan

**Affiliations:** 1 National Centre for Food Science, Singapore Food Agency, 7 International Business Park, Singapore 609919, Singapore; 2 Department of Food Science & Technology, Faculty of Science, National University of Singapore, Science Drive 2, Singapore 117542, Singapore

**Keywords:** PFAS, total dietary study, occurrence, dietary exposure, Singapore

## Abstract

Per- and poly-fluoroalkyl substances (PFAS) are synthetic compounds known for their environmental persistence, bioaccumulation, and adverse human risks. This study presents Singapore’s first Total Diet Study assessing the occurrence and dietary exposure of four PFAS, with 503 pooled samples analysed across 23 categories of commonly consumed food prepared using representative cooking methods. The majority (75%) of the food categories had non-detectable levels. Fish and seafood accounted for 89.7% of detections, with frequencies in decreasing order of PFOS > PFOA > PFNA > PFHxS, and an overall mean concentration of 0.67 µg/kg. As 93% of results are left-censored, dietary exposure assessment using a lower bound approach (LB) identified clams and crabs as having a higher estimated exposure risk for consumers, exceeding the EFSA’s Health-Based Guidance Value of 0.63 ng/kg bw/day by factors of 4.8 and 1.9, respectively. PFAS LB dietary exposure for the general population was estimated at 3.1 × 10^−3^ ng/kg bw/day (average) and 1.7 × 10^−1^ ng/kg bw/day (95th percentile), indicating a low risk to the population. However, the limit of quantification of 0.1 μg/kg requires improvement to reduce left-censored data and improve dietary estimates. Further research including a broader range of PFAS compounds is recommended for high-risk food categories to increase the accuracy of exposure assessments.

## 1. Introduction

Per- and polyfluoroalkyl substances (PFAS) are a class of synthetic molecules with fully (per) or partly (poly) fluorinated carbon chains that do not occur naturally. Possessing a wide range of unique and invaluable physicochemical properties [1], such as exceptional thermal stability, chemical inertness, surfactant capabilities, and a low coefficient of friction [2,3], PFAS have been extensively used [4] in industries and consumer products. PFAS are released into the environment during manufacturing [5], usage [6], disposal [7], or from surface runoff or accidental releases [8]. Some ionic PFAS, due to their high water solubility and environmental mobility [9], are widely dispersed through ocean currents and atmospheric transport [10]. Their strong C–F bonds make PFAS resistant to degradation and elimination, promoting bioaccumulation and biomagnification in organisms and the food chain [11]. Trophic magnification factors >1 have been observed in aquatic food webs [12]. PFAS are the most ubiquitous anthropogenic contaminants [13], with widespread detection in human blood [14] and increasing trends in exposure [15] through multiple routes, including direct surface contact, inhalation, utero exposure, breastfeeding [16], and the consumption of water and food.

Despite their benefits, PFAS are proven to be harmful to human health [17], with toxicity and epidemiology studies associating them with a variety of detrimental health effects, such as chronic liver damage [18], thyroid hormone disruptions [19,20], immunosuppression [21], and reduced vaccine efficiency [22]. PFAS research has focused on a limited subset of compounds, and the potential cumulative effects of co-exposure remain underexplored [23]. Perfluorooctanesulfonic acid (PFOS) and perfluorooctanoic acid (PFOA) are the most widely studied PFAS, with the longest production history and the most abundant evidence of their toxicities [24]. PFOA, together with other long-chain PFAS such as PFNA and PFHxS, has been added to the REACH Candidate list of Substances of Very High Concern (SVHC) [25], which identifies substances with serious and often irreversible effects on human health and the environment. The Stockholm Convention’s Persistent Organic Pollutants Review Committee (POPRC), which has listed PFOA, PFOS, and PFHxS as Persistent Organic Pollutants (POPs) [26,27,28,29], concluded that long-chain perfluorocarboxylic acids (LC-PFCAs), their salts, and related compounds are likely to cause significant adverse effects on both human health and the environment [30]. POPRC proposed restrictions on the production and use of LC-PFCAs, as well as their elimination during waste management and disposal in an environmentally sound manner [30].

There have been regulatory efforts and continuous monitoring of PFAS in food by the relevant authorities in the EU, the USA, Australia, Japan, and New Zealand. Health-based guidance values (HBGVs) have been established, but these values vary internationally. The EFSA has set maximum levels for certain food items [31] and established a tolerable weekly intake (TWI) of 4.4 nanograms per kilogram of body weight (ng/kg bw) per week, which is equivalent to a tolerable daily intake (TDI) of 0.63 ng/kg bw, for the four PFAS (i.e., PFOA, PFOS, PFNA, and PFHxS) [14]. The Agency for Toxic Substances and Disease Registry (ATSDR) of the United States [17] recommended Minimal Risk Levels (MRLs) of 3 ng/kg bw for PFOA and PFNA, 2 ng/kg bw for PFOS, and 20 ng/kg bw for PFHxS. ATSDR’s MRLs estimate daily human exposure levels to hazardous substances unlikely to cause non-cancer health effects. The Food Safety Commission of Japan (FSCJ) recommended a TDI of 20 ng/kg bw [32] for two PFAS (PFOA and PFOS). Food Standards Australia New Zealand (FSANZ) [33] recommended TDI of 160 ng/kg bw for PFOA and 20 ng/kg bw for PFOS. Biomonitoring studies have shown that four PFAS, PFOA, PFOS, PFNA, and PFHxS, account for 90.7% of total PFAS detections in adults and 87.1% in children [14]. The European Food Safety Authority (EFSA) set regulatory limits in Commission Regulation 2023/915 [31] for four PFAS and their sum for various food categories. FSANZ has established trigger points [34] for various food commodities for PFOA, PFOS, and PFHxS. This study focuses on the occurrence of four PFAS (PFOA, PFOS, PFNA, and PFHxS) in commonly consumed food in Singapore and estimates the exposure risk for the population.

Total Diet Study (TDS) is a systematic approach used to evaluate the safety of foods consumed by a population across their entire diet [35]. It involves the comprehensive analysis of representative, typically prepared local foods and beverages that are usually consumed to determine dietary exposure to foodborne hazards and to establish baseline data on contaminant levels. By assessing dietary exposure to harmful substances and conducting risk assessments, TDS contributes to improved public health outcomes by helping to ensure the safety of the food supply [35] through legislation and regulation. PFAS testing within TDS frameworks has been conducted by various agencies, including FSANZ [36], FSCJ [37], the German Federal Institute for Risk Assessment (BfR) [38], and the U.S. Food and Drug Administration (US FDA) [39]. However, there have been limited studies on PFAS exposure using TDS in Southeast Asia. In Singapore, the culinary practices and diets of the four main ethnic groups differ significantly, making it important to include their distinct food preparations in the TDS for representative dietary assessments. Different food preparation methods might affect the contaminant levels in the consumed food products.

Our previous publication [40], Singapore’s first total diet study (TDS), only reported PFOS detections in tuna and mackerel. The current work presents a detailed description of the targeted analytical method employed for the TDS. The method achieved a lower LOQ of 0.1 μg/kg, which was aligned with EFSA’s recommendation for PFAS analysis in food [14,41]. This paper presents a comprehensive profile of the occurrence data of PFAS in commonly consumed food in Singapore. It represents the first total diet study on PFAS conducted by a member nation of the Association of Southeast Asian Nations (ASEAN) and provides insight into the baseline levels of PFAS intake in the population. As a multiracial country, Singapore offers a valuable preliminary localised perspective on PFAS dietary exposure across the ASEAN population. A risk assessment was also conducted for the Singapore population and was compared with those of other countries.

## 2. Materials and Methods

### 2.1. Chemicals and Reagents

Individual PFAS standard solutions in methanol (50 µg/mL), specifically perfluorooctanoic acid (PFOA) and perfluorononanoic acid (PFNA), were obtained from Wellington (Ottawa, ON, Canada). Potassium perfluorohexane sulfonate (PFHxS) and sodium perfluorooctanesulfonate (PFOS) were sourced from Cambridge Isotope Laboratories (Tewksbury, MA, USA).

Isotopically labelled individual PFAS standards at a concentration of 50 ppm (µg/mL) in methanol, including ^13^C_8_ perfluorooctanoic acid (MPFOA), ^13^C_8_ perfluorooctane sulfonate (MPFOS), and ^13^C_9_ perfluorononanoic acid (MPFNA), were also procured from Cambridge Isotope Laboratories (Tewksbury, MA, USA).

Anhydrous sodium chloride (NaCl) and magnesium sulphate (MgSO_4_) for QuEChERS extraction were obtained from Sigma Aldrich (St. Louis, MO, USA) and Nacalai Tesque (Kyoto, Japan). PSA was sourced from Phenomenex (Torrance, CA, USA), while acid-washed, steam-activated carbon Darco G-60 came from VWR (Singapore). Formic acid (ACS grade) and 25% ammonium hydroxide were purchased from Merck (Darmstadt, Germany); LCMS-grade ammonium acetate from Merck (St. Louis, MO, USA); HPLC-grade acetonitrile (ACN) from Fulltime (Hefei, China); LCMS-grade methanol from Fisher Chemical (Fair Lawn, NJ, USA). Ultrapure water (18.2 MΩ-cm) was freshly prepared using an Elga Purelab Prima DV 35 from Veolia (High Wycombe, UK).

### 2.2. Food Sampling and Preparation

In this study, more than 4000 individual samples were acquired and processed, resulting in a total of 503 pooled samples for subsequent analysis (Table S1). The locally sourced food items were classified into 23 distinct categories based on the Codex GSFA food category system [42]. Sample preparation employed commonly used local cooking techniques identified through a population survey. These methods included reconstitution (RC), boiling (BL), soup—solids (SS), soup—liquid (SP), deep frying (DF), stir frying (SF), stewing (SW), pan frying (PF), steaming (SM), braising (BR), half-boiling (HB), baking (BK), roasting (RS), brewing (BW), grilling (GR), and serving uncooked (NC). Table 1 provides details on the number of samples per food category and their respective preparation methods. Further specifics regarding sample preparation and pooling protocols have been previously documented [40]. Following cooking, all food samples were homogenised using a food processing blender, sealed in polyethylene zip-lock bags, and stored at 4 °C until analysis.

### 2.3. Sample Preparation Method

Every new lot of containers (centrifuge tubes, glass reagent bottles, etc.), consumables (SPE, etc.), materials, and reagents used were pre-screened for the four PFAS to ensure that the levels were at least three times lower than the LOD. The experiment was conducted in a dedicated fume hood reserved for PFAS analysis to prevent contamination.

A homogenised sample portion of 1 g was weighed into a 50 mL polypropylene centrifuge tube. The sample was spiked with 50 μL of a 20 μg/kg isotopically labelled standard solution. Subsequently, 10 mL of water, 10 mL of ACN, and 150 μL of concentrated formic acid were added. The capped tube was mixed by vortex, sonicated in an ice bath for 10 min, and shaken at 250 rpm for 10 min.

For phase separation, a pre-weighed mixture of 4 g MgSO_4_ and 1 g NaCl was added. The tube was vortexed for 1 min and centrifuged at 4000 rpm at 15 °C for 10 min. The ACN layer was transferred into a new 15 mL PP centrifuge tube containing 400 mg of MgSO_4_, 300 mg of PSA, and 150 mg of activated carbon. After a minute of vortex, the tube was centrifuged at 4000 rpm, 15 °C for 10 min. The supernatant was transferred into a new 15 mL PP tube and concentrated to approximately 0.5 mL using a TurboVap LV from Biotage (Boston, MA, USA) with a gentle stream of nitrogen and a water bath at 45 °C. The concentrated solution was reconstituted to about 6 mL with ultrapure water, vortex-mixed, and centrifuged at 4000 rpm at 4 °C for 10 min.

Using an SPE manifold, the supernatant was loaded into an Oasis WAX 60 mg SPE cartridge from Waters (Wexford, WX, Ireland). The SPE cartridge was preconditioned with 3 mL of 1% ammonium hydroxide in MeOH, 3 mL of MeOH, and 3 mL of ultrapure water. With a flow by gravity, the SPE cartridge was washed with 3 mL of 2% formic acid, followed by 3 mL of MeOH, before being vacuum dried. PFAS was eluted from the SPE cartridge with 4 mL of 1% ammonium hydroxide in MeOH, collected in a new 15 mL PP tube, and dried using the TurboVap LV. The dried residue was reconstituted in 250 μL of 80% MeOH in ultrapure water, vortex-mixed, and filtered through a 0.1 μm Whatman Anatop syringe filter from Cytiva (Dassel, Germany) into a vial with a spring insert for subsequent LC-MS/MS analysis.

### 2.4. LC-MS/MS Analysis

Liquid chromatography separation was performed using an Agilent 1290 Infinity LC system from Agilent Technologies (Böblingen, BW, Germany). This system included a delay column (Gemini C18, 50 mm × 4.0 mm, 3 μm) from Phenomenex (Torrance, CA, USA) and a reverse-phase analytical column (Poroshell 120 EC-C18, 100 mm × 2.1 mm, 2.7 μm) from Agilent Technologies (Santa Clara, CA, USA). Detection was achieved using a Sciex Qtrap 5500+ quadrupole tandem mass spectrometer from AB Sciex (Framingham, MA, USA) equipped with an electrospray ionisation source. Data acquisition and processing was performed using Analyst 1.7.1 from AB Sciex (Framingham, MA, USA). The mobile phase consisted of 1.25 mM ammonium acetate in ultrapure water (Eluent A) and MeOH (Eluent B). The column compartment temperature was set at 45 °C, and the injection volume was 5 μL. A flow rate of 0.3 mL/min was used for the gradient elution programme: 5% B at 0 min, increasing to 40% B at 2 min, then to 95% B at 18 min. The gradient was held at 95% B for 2 min before decreasing back to 5% B at 20.1 min and holding until 25 min.

The MS/MS detection used a multiple reaction monitoring (MRM) scan type in negative polarity mode. The ion source temperature (TEM) was set at 350 °C, curtain gas (CUR) at 35, nebulizing gas (GS1) at 40, drying gas (GS2) at 40, and entrance potential at 10. The optimised compound-dependent parameters, such as precursor and product ions and collision energies, are presented in Table 2. Due to the lack of availability of internal standards from vendors for PFHxS at the time of analysis, MPFOA was used as the internal standard for PFOA.

For quality assurance, each batch was analysed by checking spiked samples for recoveries between 80% and 120%, running a single reagent blank prepared with the batch and correcting sample values accordingly, and injecting the mid-point calibration standard after every nine samples to confirm that instrument accuracy was within 90% to 110% and sensitivity did not drift significantly. A methanol injection was performed before each mid-point calibration standard to check for carryover. The laboratory regularly participated in international interlaboratory exercises (proficiency tests), and measurement uncertainty was used for decisions. No PFAS analytes were detected above the LOQ for the blanks.

### 2.5. Method Validation

The method was validated across three food categories, meat, vegetables, and carbohydrates, using three representative matrices per category (Table A1). The method was validated within a calibration range from 0.1 μg/kg to 2 μg/kg, yielding good recoveries of 86% to 115%. Both matrix-matched and external calibration curves exhibited coefficients of determination (R^2^) greater than 0.99. Repeatability within each food category, assessed by triplicate analyses at low (0.4 μg/kg), middle (0.8 μg/kg), and high (1.2 μg/kg) spike levels, remained below 4.8%. Intermediate precision, evaluated by two analysts on separate days, did not exceed 5.8%. Limits of detection (LOD) and quantitation (LOQ) are calculated as 3.3 and 10 times the standard error of the y-intercept divided by the slope from the matrix-matched calibration curve; all validated matrices demonstrated LODs below 0.03 μg/kg and LOQs below 0.1 μg/kg. Relative expanded uncertainties are under 5.3%. Use of MPFOA for PFHxS produces a bias of −6.2%. To confirm applicability across diverse real-world samples, an additional 15 food matrices (Table A1) were spiked at the mid-level (0.8 µg/kg), producing recoveries of 87% to 111% for PFOA, PFNA, and PFOS, and 83% to 118% for PFHxS. This underscores the method’s robustness and demonstrates that the use of MPFOA as an internal standard for PFHxS produces acceptable recoveries.

### 2.6. Food Consumption Data

Twenty-four-hour dietary recall surveys [40], categorised into general consumers and high consumers (i.e., 95th percentile, P95), were conducted by the Singapore Food Agency (SFA) between 2021 and 2022 to gather food consumption data from the Singapore population. A total of 2014 participants, aged between 15 and 92 years, were surveyed twice for consumption patterns on both a weekday and a weekend. The survey group, stratified by age, ethnicity, and gender, was representative of the Singapore population. The mean intake of each food product was calculated by averaging each respondent’s consumption statistics. “Consumers” refers to individuals who consume a food category, whereas “population” includes both consumers and non-consumers of that category.

### 2.7. Dietary Exposure Assessment

A deterministic approach was used to estimate the dietary exposure to PFAS of an average consumer (μg/kg bw/day) (Equation (1))

(1) $$Dietary\;Exposure=\sum_{i=1}^{n}\left(C_{i}\times E_{i}\right)$$

where *C* is the mean occurrence concentration of the PFAS in each food sample (ng/kg), denoted as *i*, and *E* is the daily consumption data in each of the food samples per kilogram body weight of an individual (kg/kg bw day); *n* is the number of food samples in each food category.

The hazard quotients (HQs) for the four PFAS were calculated by dividing the dietary exposure values by their corresponding HBGVs (expressed as ng/kg bw day), as specified in Equation (2).

(2) $$Hazard\;Quotients\;(HQs,\;unitless)=\frac{Estimated\;dietary\;exposure}{Tolerable\;Daily\;Intake\;(TDI)}$$

## 3. Results and Discussion

### 3.1. Occurrence of PFAS in Food

In this study, the most frequently detected PFAS across the food categories involving various preparation methods were PFOS, followed by PFOA, PFNA, and the least detected being PFHxS. Their percentage contributions, detection rates, and mean occurrence values are summarised in Table 3. The order of detection for the four PFAS in this study was also observed in China’s sixth TDS [43]. PFOS was also reported as the most frequently detected PFAS by the US FDA [44] and FSANZ [36] in their TDS. The overall percentage contributions of each PFAS in this study are comparable to those reported by EFSA [14]. A majority (75%) of the food categories showed non-detectable levels (ND; <LOD of 0.03 μg/kg), with only a limited number of samples exceeding the level of quantification (LOQ; ≥0.1 μg/kg), resulting in 93% of the dataset being left-censored. A lower-bound approach (LB) was adopted to handle the left-censored low-level contaminant dataset, referencing guidelines from the World Health Organization (WHO) and the Food and Agriculture Organization of the United Nations (FAO) [45], whereby values below the LOQ were assigned a value of zero. Exposure assessment using LB values, rather than upper-bound approach (UB) levels—which apply LOQ or LOD to non-detected data—is considered more realistic when a high proportion of results are left-censored. This approach is supported by the EFSA CONTAM Panel during their PFAS exposure assessment [14]. In this study, the middle-bound approach (MB) and UB are provided for reference. For values below the LOQ, the MB calculation will replace the value with 0.03 μg/kg (LOD), whereas the UB calculation will replace the value with 0.1 μg/kg (LOQ). Given the high proportion of left-censored data (93%), the mean was used to calculate occurrence values instead of the median, as the latter would result in a value of zero.

Four food categories were detected with occurrence values above LOQ, namely, “eggs and egg products” (EP), “sauces and condiments” (SC), “fish and seafood” (FS), and “meat and meat products” (MP). Similarly, three food categories (FS, EP, and MP) were also reported by the EFSA to be major exposure contributors for PFOS and PFOA [14]. The detection rates and mean LB occurrence values are listed in Table 4.

**Figure A1:**
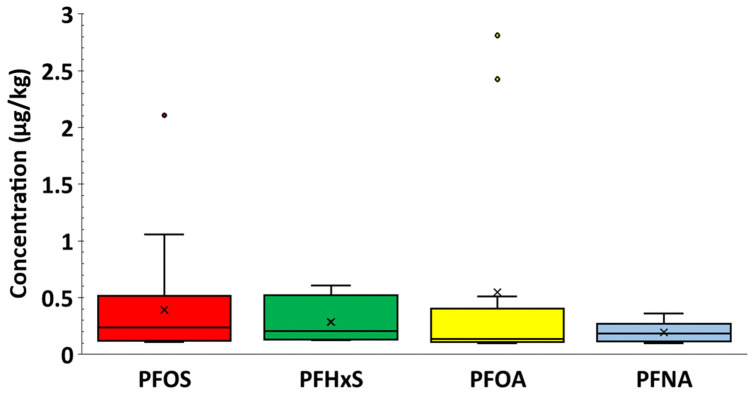
Box-and-whisker plot illustrates the distribution of occurrence values for the four PFAS compounds analysed in this study. The interquartile range (IQR) is depicted by the bars, with the bottom and top representing the lower quartile (Q1, 25th percentile) and upper quartile (Q3, 75th percentile), respectively. Whiskers extend from the bars with minimum and maximum values that are 1.5 times the IQR from the quartiles. Crosses and dots indicate mean occurrence values and outliers respectively, whilst the line within the box indicates the median.

PFAS detection (≥LOD) in this study is primarily from the fish and seafood category (89.7%) with minor contributions from other food categories, such as eggs (5.8%) and meat products (1.9%). The values above LOQ for PFOA, PFOS, PFNA, and PFHxS for fish and seafood were between 0.10 and 2.81 μg/kg, 0.11 to 2.11 μg/kg, 0.10 to 0.36 μg/kg, and 0.13 to 0.61 μg/kg, respectively. The values above LOQ for PFOA (0.11 to 0.15 μg/kg) and PFOS (0.11 to 2.11 μg/kg, with 0.11 to 0.64 μg/kg found when excluding anchovies) in fish were comparable to values in the literature found in Vietnam, with PFOA ranging from 0.01 to 0.19 μg/kg, while PFOS ranged from 0.03 to 0.48 μg/kg [46]. The distribution of PFAS values above LOQ for the four PFAS, depicted in Figure A1, might seem different (RSD ranging from 47 to 173%), but there is no significant difference at a 99% confidence level between the mean of the detected values using the Kruskal–Wallis test (Table S2). This demonstrates that, whilst the detection rates of the four PFAS differ (Table 3), the mean concentration of values above LOQ is statistically similar once the compounds are present.

The detection rates of PFOA and PFOS for meat and meat products in this study are lower compared to the EFSA report [14] (Table 4) and are comparable for PFNA and PFHxS. The detection rates of the individual PFAS for fish and seafood in this study differed from those in the EFSA report [14] (Table 4) but were comparable for PFNA and PFHxS. This study observed higher detection rates for the four types of PFAS across most categories compared to findings reported by the EFSA. This difference is attributed to the lower LOD of 0.03 μg/kg in the current analysis, as opposed to the EFSA’s LOD of 0.30 μg/kg. Despite this, the PFOS mean level in Singapore’s seafood was notably low, being almost 20% of the level in the EFSA report. This is likely because the fish and seafood in Singapore are mostly imported from neighbouring countries or produced from local farms, and there have been no large-scale PFAS manufacturing activities [47].

In the fish and seafood category, 31 sample types were analysed, and the detected PFAS concentrations are presented in Table A2, with details on the detection rates for individual PFAS compounds. Table A4 summarises the mean occurrence levels and standard deviations across various fish and seafood samples. Within this category, PFOS was the most frequently detected PFAS, followed by PFNA, PFOA, and PFHxS, with detection rates of 28.0%, 17.2%, 14.0%, and 4.3%, respectively. The corresponding average concentrations were 0.39 μg/kg, 0.19 μg/kg, 0.55 μg/kg, and 0.29 μg/kg.

**Figure 1:**
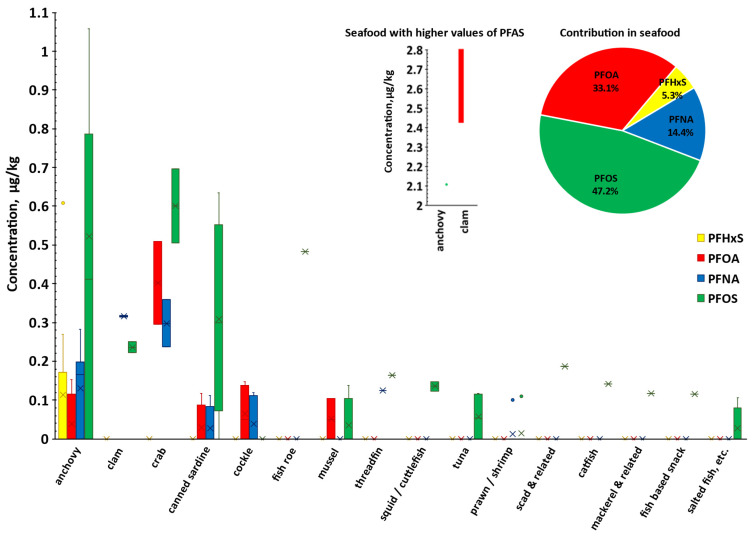
The box-and-whisker plot presents the concentration (μg/kg) distribution of individual PFAS for the 31 types of seafood tested. For anchovies and clams, values greater than 1.10 μg/kg are depicted in the smaller plot. The interquartile range (IQR) is depicted by the bars, with the bottom and top representing the lower quartile (Q1, 25th percentile) and upper quartile (Q3, 75th percentile), respectively. Whiskers extend from the bars with minimum and maximum values that are 1.5 times the IQR from the quartiles. Crosses and dots indicate mean occurrence values and outliers respectively, whilst the dash indicates the median. The accompanying pie chart illustrates the relative contribution of each PFAS to the total PFAS burden on fish and seafood.

The pie chart in Figure 1 shows the proportion of each PFAS in the total concentration found in fish and seafood, reflecting the pattern seen in Table 4. Clams, crabs, canned sardines, and anchovies had the highest detection rates and average levels among the seafood samples. A comparison of individual PFAS levels in various seafood types with FDA seafood study data [48] indicates that, although clams exhibited notably elevated PFOA concentrations (ranging from 2.43 to 2.81 μg/kg; see Figure 1), these values remain below the FDA-reported range of 3.0 to 20 μg/kg. The observed PFOS concentrations in clams (0.22 to 0.25 μg/kg) also fell within the lower end of the FDA report’s [48] range (0.19 to 1.23 μg/kg). For crabs, the measured PFOA levels (0.09 to 0.51 μg/kg) and PFNA levels (0.05 to 0.35 μg/kg) in this study were consistent with those reported by the FDA [48], whereas PFOS concentrations exceeded the FDA detection range of 0.07 to 0.39 μg/kg. Additionally, anchovies and sardines showed significantly higher PFOS concentrations (0.29 to 2.11 μg/kg) than the maximum level of 1.15 μg/kg previously reported in the literature [49].

The wide range of PFAS values observed across different seafood sources (Figure 1) indicates substantial variability in contamination levels, highlighting the need for ongoing monitoring in this food group.

The anchovies analysed in this study were small, whole, dried anchovies with viscera intact, reflecting the predominant form consumed locally, which accounts for over 91% of imported anchovies. Viscera have been shown in the literature [50] to contain higher levels of PFAS compared to the fillet. The drying process, which typically results in a 37–60% reduction in weight [51], potentially contributes to the concentration of PFAS within the dried anchovies.

Within the sauces and condiments category, sambal chilli was the only sample found to contain PFAS. The preparation of sambal chilli typically includes seafood components such as anchovies and prawn paste, along with other ingredients like chilli, garlic, ginger, sugar, salt, and lime. In our study, these individual plant-based ingredients showed no detectable levels of PFAS. However, the anchovy and prawn samples tested positive, with the sum of their PFAS concentrations ranging from 0.50 to 3.12 μg/kg and 0.20 to 0.21 μg/kg, respectively. Therefore, the presence of PFAS in sambal chilli is highly likely to be attributable to the bioaccumulation of these compounds in the seafood components, identifying them as the probable primary source of PFAS in this category.

All PFAS detections in the egg and egg product category come from preserved duck eggs, like century eggs (the majority of PFAS levels < LOQ with a single detection of PFOA at 0.15 μg/kg) and salted duck eggs (levels < LOQ), rather than hen eggs. The main destination of egg exports from Malaysia is Singapore [52], and a study from Malaysia [53] also found PFOA and PFOS in duck eggs at levels (PFOA < 0.10 while PFOS < 0.50 μg/kg) that align with what was found in this study. Despite employing lower limits of detection that yielded higher PFAS detection rates in eggs compared to the EFSA [14], the mean sum of the four PFAS concentrations (0.15 μg/kg) measured in this study was lower than those reported by the EFSA (0.38 μg/kg).

Pork organs, and no other meat products, are the source of all PFOS detections in the meat category in this study. Literature has shown that edible offal from animals has much higher levels of PFAS as compared to non-offal meat cuts [54,55]. The Federal Agency for the Safety of the Food Chain (FASFC) has estimated that PFOS concentrations in bovine liver are, on average, eight times higher than those in the muscle tissue of the same animal and concluded that organs are higher-risk food items [56]. As this study tested only one composite organ sample from pork, found to have 0.13 μg/kg of PFOS, further research on various offal types across different animal species is needed to conduct a comprehensive risk assessment of PFAS for offal consumers.

### 3.2. Dietary Exposure to PFAS

Using Equation (1), the mean LB occurrence values from Table 4, along with consumption data from dietary surveys, were used to estimate dietary exposure for the general population and for consumers (Table A3). As recommended by the EFSA [14], due to the high left-censored results, exposure calculations using MB and UB should be considered to be only a rough indication of the range of chronic dietary exposure and should therefore be interpreted with caution. LB dietary exposure values will be used for risk assessment discussions, as they are more realistic. In the Singapore population, the LB mean estimated dietary exposure to these four PFAS is 3.1 × 10^−3^ ng/kg bw/day, substantially lower than the values reported for PFOA and PFOS in China’s sixth Total Diet Study (7.0 × 10^−1^ ng/kg body weight per day) [43] and in Japan’s 2012–2014 Total Diet Study (6.6 × 10^−1^ ng/kg body weight per day) [37].

EFSA CONTAM established the TWI using physiologically based pharmacokinetic (PBPK) modelling and based on the benchmark dose level (BMDL10) of 17.5 µg/L from the observed adverse effects of the four PFAS on the infant immune response [14]. The TWI equates to a tolerable daily intake (TDI) of 0.63 ng/kg bw per day. This study uses the EFSA’s TDI, the most stringent of the available HBGVs, along with values from Table A3 to evaluate the risk of PFAS exposure through food consumption, using Equation (2) to calculate HQs. The results are presented in Table 5, in which HQ values that are significantly below one indicate that the estimated exposure is much lower than the TDI and is therefore generally considered safe. The sum values for the mean and P95 HQ values for the population are very much lower than one, regardless of LB, MB, or UB substitution methods. However, the sums of the values for mean consumers’ UB HQ, and P95 consumers’ MB and UB HQ values exceed 1, whilst their respective LB HQ values are much lower than 1. It is more likely that the true HQ value lies between LB and MB, whilst the UB values are likely overestimates due to the high method LOQ. Instead of weekly consumption data, a 24 h dietary survey was conducted for consumption patterns on both a weekday and a weekend in different months. The use of TDI for HQ calculations conservatively assumes identical consumption throughout the week.

For the dietary exposure assessment in Singapore, a worst-case scenario assumes that an individual consumes all PFAS-contaminated food items across the four categories listed in Table 5. Under this scenario, the exceedance of the EFSA’s TDI (0.63 ng/kg bw per day) ranges from 0.2 times (for LB mean consumption rate) to 0.5 times (for LB 95th percentile consumption rate). Even with UB, the exceedance of the EFSA’s TDI is from 1.02 times (for UB mean consumption rate) to 2.59 times (for UB 95th percentile consumption rate), which is lower compared to German adults’ PFAS TDS exposure assessment [38] of 2 (LB, Mean) to 5 times (LB, P95). BfR monitored data from 11 food categories, all of which are included in this study. Singapore’s estimated population mean dietary exposure (LB and UB) to the four PFAS is lower compared to those reported [57] in Belgium, the Czech Republic, Italy, and Norway.

Among the four food categories with quantifiable PFAS occurrence, the most significant intake of PFAS is from the fish and seafood category, which aligns with the conclusions reported by the EFSA [14] and FDA TDS [58]. The contribution order observed in Table 5 mirrored the EFSA’s dietary exposure assessment for PFOA and PFOS [14], which identified fish and seafood as the predominant contributors, followed by eggs and meat. For the mean and 95th percentile of the general population (Table 5) for all three bound approaches, PFAS exposure from dietary intake of other food categories, such as eggs and egg products, sauces and condiments, and meat and meat products, is significantly lower than the EFSA TDI limit. However, for P95 consumers of fish and seafood, the HQ ranges from 0.40 (LB) to 0.92 (UB), which exceeds the bulk of the quota of 1, indicating a potential cause of concern.

In the eggs and egg products category, both mean and P95 consumers exhibited HQs well below 1 relative to the EFSA HBGV. Hen eggs, which were the most consumed egg product, were eaten by 14.3% of respondents, whereas only 2% reported consuming preserved duck eggs, indicating a low risk of PFAS exposure from the latter. However, given the limited literature on PFAS in preserved duck eggs, further research is needed. Similarly, the meat and meat products category demonstrated low HQs. According to the consumption survey [40], only 1% of participants consumed pork organs, a figure significantly lower than that for other animal products, suggesting a low risk of PFAS exposure in this category. This finding contrasts sharply with China’s sixth TDS [43], which identified eggs and meat as the primary contributors to PFOA intake, and PFOA alone represented a HQ of 0.5 for the mean population.

The EFSA’s report [14] indicated that the four PFAS (PFOA, PFOS, PFNA, and PFHxS) accounted for approximately 50% of the LB exposure out of the 17 PFAS analysed. Therefore, as in the EFSA’s conclusion, there could be a potential bias of 50% or more in this study regarding the actual dietary exposure to all PFAS.

### 3.3. Dietary Intake of PFAS from Fish and Seafood

This study identified notably higher levels of PFAS in fish and seafood compared to other food categories. In Singapore, the average annual per capita consumption of fish and seafood is 22 kg [59], above the global average of 20.5 kg [60]. As a result, dietary exposure assessment for PFAS from fish and seafood is particularly relevant.

A comprehensive analysis of dietary PFAS exposure from various seafood types is presented in Table A5, with Table 6 summarising the percentage of the EFSA’s TDI represented through consumption of each individual food category. In a scenario where an individual consumes all listed seafood categories, the combined hazard quotients (HQs) exceed the TDI by factors ranging from 9.72 (mean, LB) to 24.1 (P95, LB). Notably, clams and crabs contributed over 60% of both the mean and P95 LB HQs for consumers in the seafood category, underscoring the significant health risks associated with these items. Mean consumption of clam and crab is currently 0.930 g/kg bw and 0.929 g/kg bw, respectively. Mean consumers of clam and crab need to reduce their consumption by 79% and 48%, respectively, to achieve an HQ (LB) of 1, and reduce their consumption by 89% and 74%, respectively, to achieve an HQ (LB) of 0.5. Moreover, the P95 consumers (LB) of canned sardines and catfish experience exposures surpassing the TDI by 2.06 and 1.77 times, respectively. These findings highlight the need for targeted risk management strategies, including consumer advisories and increased public education, to address potential health impacts.

As expected for the more frequently detected seafood categories (clam and crab), the HQ values from the different bounded approaches (LB, MB, UB) produce similar values. However, for highly consumed types of seafood, such as mackerel, the HQ value from the UB approach can differ significantly from the HQ value from the LB approach. Improvements to the method’s LOQ can greatly reduce the range of values between the LB and UB approaches.

**Figure 2:**
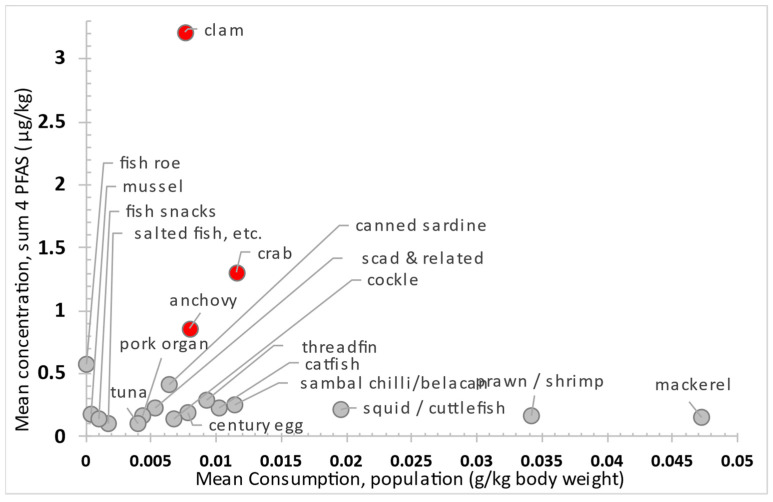
Scatter plot of mean consumption versus mean occurrence concentration (LB) across food categories, illustrating relative risk. Items with elevated risk profiles are highlighted in red to denote higher exposure potential.

Figure 2’s scatter plot shows mean consumption versus mean PFAS levels in seafood. Clams, crabs, and anchovies fall in the upper-left quadrant, which suggests higher exposure and risk. Our food consumption survey [40] found low consumption rates for clams (0.8%), crabs (1.2%), and canned sardines (0.7%), but anchovies are more commonly eaten (7.4%). Despite the high PFAS concentrations in clams, crabs, and sardines, their limited intake contrasts with frequent anchovy consumption. Reducing consumption of seafood with high PFAS, especially among frequent consumers, can help lower health risks.

### 3.4. Cooking Method on PFAS Concentration

Regarding the impact of cooking methods on PFAS concentrations, the levels observed (Table A6) change slightly across different techniques. Minor variations in PFAS concentrations across the different cooking methods may be attributed to the differences in weight loss from moisture loss during frying [61] or moisture gain in soup solids. ANOVA tests (Table S3) for anchovy (PFOS and PFNA) and cockle (PFOA and PFNA) showed no significant differences between the means from the different cooking methods. There is a general trend of increasing PFAS concentration with greater cooking intensity, possibly resulting from the reduction in food mass associated with more intense cooking processes, or from the increased tendency for precursor PFAS to decompose into legacy PFAS. Conducting sum parameter analysis, such as non-targeted PFAS analysis, can help identify other potential organofluorine compounds that may contribute to legacy PFAS concentrations across different cooking processes. Interestingly, although deep frying is a more intense cooking method than pan frying, PFOS levels in deep-fried anchovies were lower than those in pan-fried samples. Contrary to the expectation that higher moisture content correlates with lower PFAS levels, boiled squid and cuttlefish exhibited higher PFOS concentrations than their stir-fried counterparts. These findings suggest that cooking methods affect PFAS concentrations inconsistently across seafood species, cooking methods, and PFAS compounds. This conclusion has also been reported in the literature [61]. An interesting observation was seen for anchovy soup, where all measured PFAS remained in the solid anchovy rather than migrating into the broth. This phenomenon may be attributed to the tendency of long-chain PFAS to preferentially bind to organic material rather than dissolve in aqueous solutions. This finding could potentially be beneficial for consumers of Chinese cuisine, as many chefs prepare broths and soups using anchovies and subsequently discard the solid fish.

Cockles are sometimes consumed partially cooked, so they were analysed in their raw form. They were the only seafood in which PFAS were detectable (Table A6) in both the uncooked and cooked state. Comparison of raw, boiled, and stir-fried samples showed that cooking was observed to increase the concentrations of the dominant PFAS (PFOA and PFNA) in cockles. This increase may be attributed to weight reduction during cooking, as reported in a previous study [61].

### 3.5. Limitations of Study and Future Improvements

Several limitations were identified in the current study.

- The LOQ of 0.1 μg/kg still resulted in a high number of left-censored data points, limiting the precision of risk assessment. Improvements in the analysis method are necessary to achieve lower detection limits for more accurate exposure assessment. Commission Recommendation (EU) 2022/1431 [62] proposed LOQ for the four PFAS compounds: 0.01 μg/kg for milk and 0.005 μg/kg for fruits, vegetables, roots, tubers, and wild fungi.
- The Commission recommended analysing 24 other PFAS compounds where feasible. Unlike non-targeted analysis, which could perform a retrospective analysis, the current targeted analysis limits our analysis to the four PFAS. In addition to the four PFAS, the detection of other PFAS such as perfluorodecanoic acid (PFDA) and perfluoroheptanoic acid (PFHpA) in a Singaporean biomonitoring study [63] highlights the necessity of expanding food surveillance programmes to encompass a broader spectrum of PFAS and to employ an analytical method with sufficiently low detection limits for accurate exposure assessment. Future work should also include perfluorobutanoic acid and perfluorohexanoic acid, since the EFSA’s report [14] indicated that these two PFAS accounted for the other 50% of the LB exposure, in addition to PFDA and PFHpA. However, there is currently a lack of HBGVs for PFAS compounds beyond the four commonly assessed, which hinders the risk assessment process.
- The 24 h dietary recall used to assess food consumption patterns reflected intake only at the time of data collection and did not capture seasonal or long-term food consumption trends. This also limited the risk assessment calculation to the TDI index rather than the TWI, which the EFSA recommends for evaluating cumulative chronic exposure. Incorporating additional dietary surveying methods [64], such as food diaries, food frequency questionnaires, and repeated 24 h dietary recalls, to reflect within-week variability would provide a more comprehensive and representative estimate of dietary intake within the local population.
- Some of the food categories had small sample sizes (*n* < 10), which may not adequately reflect consumption patterns across the broader population. Because the tested samples were pooled to reduce the total number of individual analyses, the ability to trace PFAS levels in specific food items back to their original product or source country was lost. Pooling also reduces the product-level variance and inhibits hotspot detection. This highlights the need for further studies of individual samples without pooling, especially for the higher-risk seafood categories such as clams, crabs, mackerel, and anchovies.

## 4. Conclusions

This total dietary study investigated the prevalence and occurrence levels of PFAS in locally consumed food products subjected to different cooking methods. Cooking methods affected the PFAS levels in food. PFAS exposure in the Singapore population identified seafood as the primary contributor to PFAS intake. While the general population exhibited low-risk exposure levels, with intake values below the EFSA TDI values, a potential health risk was observed among average consumers of clams and crabs, as well as high consumers of canned sardines and catfish. Reducing consumption of these high-risk seafood items would lower overall PFAS exposure. Additionally, the LOQ of the current analytical method was still inadequate, resulting in a high proportion (90%) of left-censored data. To improve the accuracy of future risk assessments in total dietary studies and targeted investigations, a more sensitive analytical method capable of detecting a broader spectrum of PFAS compounds should be developed.

## Figures and Tables

**Table 1:** Cooking or preparation methods for the various food categories and the sample size (*n*).

Food Category	*n*	Cooking/Preparation Method
Fruit and fruit products	57	NC
Grains and grain products	33	NC, BL, SS, SP, SM, and SF
Beverages and bottled water	21	NC, RC, and BW
Legumes, Nuts, and Seeds	17	NC, BL, SF, PF, and DF
Eggs and egg products	15	NC, BL, PF, BR, HB, SM, and SP
Sauces and condiments	22	NC, BL, and SF
Bakery products	14	NC and DF
Infant food products	11	NC and RC
Tap and drinking water	1	NC
Stalk, stem, and bulb vegetables	26	BL, SF, SP, SW, SS, and SM
Fats and oils	7	NC, DF, SF, and DO
Composite foods	6	NC and BL
Fungi and seaweed	15	NC, BL, SS, SP, and SF
Fish and seafood	93	NC, PF, SP, SS, SF, SM, DF, BL, BR, SW, BK, and ST
Brassica vegetables	9	SF, BL, SM, and PF
Fruiting vegetables	21	SF, BL, and SM
Milk and dairy products	10	NC and RC
Ready to eat savouries	3	NC
Confectionary	6	NC
Meat and meat products	53	NC, BL, DF, BK, PF, RS, SF, SW, GR, SM, SP, SS, and BR
Root and tubers	22	BL, SF, RS, BK, SS, DF, SP, and SM
Leafy vegetables and herbs	35	SF, BL, SP, and SS
Vegetable protein	6	BR, SF, and DF

**Table 2:** The MS/MS compound parameters. For each analyte, the first line of the ion pair is the quantifier, while the second line of the ion pair is the qualifier.

Analyte	Precursor Ion (*m*/*z*)	Product Ion (*m*/*z*)	Collision Energy (CE)	Internal Standard
PFOA	413	369	15	MPFOA
		169	26	
PFOS	499	80	101	MPFOS
		99	99	
PFNA	463	419	16	MPFNA
		219	24	
PFHxS	399	80	90	MPFOA
		99	41	
MPFOA	421	376	16	-
		172	26	
MPFOS	507	80	103	-
		99	98	
MPFNA	472	427	16	-
		223	24	

**Table 3:** Profile of PFAS detection and levels in food.

All Food	PFOA	PFOS	PFNA	PFHxS	4 PFAS
Overall contribution (>LOD)	33.0%	47.8%	14.0%	5.2%	-
EFSA contribution (>LOD)	21%	66%	4%	10%	-
Quantifiable (≥LOQ)	2.8%	5.6%	3.2%	0.8%	7.0%
LB Mean values ^#^	0.01 ± 0.17	0.02 ± 0.13	0.01 ± 0.04	2.3 × 10^−3^ ± 0.03	0.04 ± 0.28
MB Mean values ^#^	0.04 ± 0.17	0.05 ± 0.13	0.04 ± 0.03	0.03 ± 0.03	0.16 ± 0.27
UB Mean values ^#^	0.11 ± 0.16	0.12 ± 0.12	0.10 ± 0.02	0.10 ± 0.02	0.43 ± 0.24
Mean for results above LOQ ^#^	0.52 ± 0.90	0.38 ± 0.41	0.19 ± 0.09	0.29 ± 0.22	0.63 ± 0.87

^#^ Mean values are expressed in μg/kg. EFSA [14] overall contribution is included for comparison. LOD of 0.03 μg/kg and LOQ of 0.1 μg/kg.

**Table 4:** PFAS sample size (*n*), detection rate, and mean occurrence values (μg/kg) in food.

Food Category	*n*	Statistic	PFOA	PFOS	PFNA	PFHxS	4 PFAS
Fish and seafood	93	Detection	39%	51%	49%	10%	68%
		Mean (LB)	0.08 ± 0.39 *	0.11 ± 0.28 *	0.03 ± 0.08 *	0.01 ± 0.07 *	0.23 ± 0.61 *
		Mean (MB)	0.10 ± 0.38	0.13 ± 0.28	0.06 ± 0.07	0.04 ± 0.07	0.33 ± 0.59
		Mean (UB)	0.16 ± 0.37	0.18 ± 0.26	0.12 ± 0.05	0.10 ± 0.02	0.43 ± 0.24
Sauces and condiments	18	Detection	0%	5%	0%	0%	5%
		Mean (LB)	0.00 ± 0.00	0.01 ± 0.05	0.00 ± 0.00	0.00 ± 0.00	0.01 ± 0.05
		Mean (MB)	0.03 ± 0.00	0.04 ± 0.05	0.03 ± 0.00	0.03 ± 0.00	0.13 ± 0.05
		Mean (UB)	0.10 ± 0.00	0.11 ± 0.03	0.10 ± 0.00	0.10 ± 0.00	0.41 ± 0.03
Eggs and egg products	14	Detection	27%	20%	13%	0%	27%
		Mean (LB)	0.00 ± 0.00	0.00 ± 0.02	0.00 ± 0.00	0.00 ± 0.00	0.00 ± 0.02
		Mean (MB)	0.04 ± 0.03	0.03 ± 0.00	0.03 ± 0.00	0.03 ± 0.00	0.13 ± 0.03
		Mean (UB)	0.10 ± 0.01	0.10 ± 0.00	0.10 ± 0.00	0.10 ± 0.00	0.40 ± 0.01
Meat and meat products	53	Detection	0%	4%	2%	0%	4%
		Mean (LB)	0.00 ± 0.00	0.00 ± 0.02	0.00 ± 0.00	0.00 ± 0.00	0.00 ± 0.02
		Mean (MB)	0.03 ± 0.00	0.04 ± 0.05	0.03 ± 0.00	0.03 ± 0.00	0.13 ± 0.05
		Mean (UB)	0.10 ± 0.00	0.11 ± 0.03	0.10 ± 0.00	0.10 ± 0.00	0.41 ± 0.03
(EFSA) Fish and seafood ^#^	2524	Detection	12.2%	37.4%	6%	1.8%	-
		Mean (LB)	0.36	2.05	0.08	0.01	-
(EFSA) Eggs and egg products ^#^	174	Detection	8%	8%	0%	3%	-
		Mean (LB)	0.11	0.27	-	0.00	-
(EFSA) Meat and meat products ^#^	169	Detection	4%	7%	1%	0%	-
		Mean (LB)	0.03	0.03	0.00	-	-

* For food categories having more than one quantifiable sample, standard deviation values are provided. ^#^ EFSA [14] fish and seafood detection (≥LOD of 0.10 μg/kg, %) is included for comparison.

**Table 5:** The mean HQs of the four PFAS were calculated for both the mean and 95th percentile (P95) consumption rate across the four detected food categories for the general population and consumers using the dietary exposure values from Table A3. Values listed are from LB | MB | UB.

	HQs for Mean		HQs for P95	
Food Category	Population	Consumers	Population	Consumers
Fish and seafood	2.69 × 10^−3^ | 3.58 × 10^−3^ | 6.15 × 10^−3^	0.16 | 0.20 | 0.33	1.01 × 10^−2^ | 1.37 × 10^−2^ | 2.32 × 10^−2^	0.40 | 0.54 | 0.92
Sauces and condiments	1.30 × 10^−4^ | 1.36 × 10^−3^ | 4.23 × 10^−3^	3.66 × 10^−3^| 3.58 × 10^−2^| 0.11	7.02 × 10^−4^ | 9.62 × 10^−3^ | 2.98 × 10^−2^	1.57 × 10^−2^ | 0.09 | 0.28
Eggs and egg products	3.10 × 10^−4^ | 4.55 × 10^−3^ | 1.46 × 10^−2^	1.28 × 10^−2^ | 7.75 × 10^−2^ | 0.24	7.71 × 10^−4^ | 2.79 × 10^−2^ | 9.07 × 10^−2^	3.32 × 10^−2^ | 0.19 | 0.56
Meat and meat products	1.35 × 10^−5^ | 3.74 × 10^−3^ | 1.24 × 10^−2^	2.30 × 10^−3^| 0.10 | 0.34	8.28 × 10^−5^ | 1.81 × 10^−2^ | 6.03 × 10^−2^	4.52 × 10^−3^ | 0.25 | 0.83
Sum	3.14 × 10^−3^ | 1.32 × 10^−2^ | 3.74 × 10^−2^	0.17 | 0.42 | 1.02	1.16 × 10^−2^ | 6.94 × 10^−2^ | 0.20	0.46 | 1.07 | 2.59

**Table 6:** The mean HQs of each seafood category from LB | MB | UB.

	HQs for Mean ^a^		HQs for P95 ^b^	
Seafood Category ^c^	Population	Consumers	Population	Consumers
Clam	3.90 × 10^−2^ | 3.90 × 10^−2^ | 3.97 × 10^−2^	4.75 | 4.75 | 4.83	1.04 × 10^−1^ | 1.04 × 10^−1^ | 1.06 × 10^−1^	12.6 | 12.6 | 12.9
Crab	2.38 × 10^−2^ | 2.44 × 10^−2^ | 2.57 × 10^−2^	1.92 | 1.96 | 2.07	4.98 × 10^−2^ | 5.09 × 10^−2^ | 5.36 × 10^−2^	4.01 | 4.10 | 4.32
Anchovy	1.09 × 10^−2^ | 1.14 × 10^−2^ | 1.28 × 10^−2^	1.47 × 10^−1^ | 1.53 × 10^−1^ | 1.71 × 10^−1^	6.37 × 10^−2^ | 6.66 × 10^−2^ | 7.46 × 10^−2^	4.05 × 10^−1^ | 4.23 × 10^−1^ | 4.74 × 10^−1^
Mackerel and Related	1.12 × 10^−2^ | 1.57 × 10^−2^ | 3.13 × 10^−2^	3.37 × 10^−1^ | 4.73 × 10^−1^ | 9.48 × 10^−1^	2.20 × 10^−2^ | 3.09 × 10^−2^ | 6.19 × 10^−2^	6.65 × 10^−1^ | 9.34 × 10^−1^ | 1.87
Threadfin	4.25 × 10^−3^ | 5.13 × 10^−3^ | 7.18 × 10^−3^	4.08 × 10^−1^ | 4.92 × 10^−1^ | 6.89 × 10^−1^	6.83 × 10^−3^ | 8.25 × 10^−3^ | 1.15 × 10^−2^	6.55 × 10^−1^ | 7.91 × 10^−1^ | 1.11
Squid/Cuttlefish	6.69 × 10^−3^ | 7.62 × 10^−3^ | 1.35 × 10^−2^	1.50 × 10^−1^ | 1.71 × 10^−1^ | 3.02 × 10^−1^	5.85 × 10^−3^ | 6.67 × 10^−3^ | 1.18 × 10^−2^	4.57 × 10^−1^ | 5.20 × 10^−1^ | 9.20 × 10^−1^
Canned sardine	4.15 × 10^−3^ | 4.76 × 10^−3^ | 6.51 × 10^−3^	6.91 × 10^−1^ | 7.92 × 10^−1^ | 1.08	1.24 × 10^−2^ | 1.42 × 10^−2^ | 1.94 × 10^−2^	2.06 | 2.36 | 3.24
Catfish	3.74 × 10^−3^ | 4.23 × 10^−3^ | 7.21 × 10^−3^	5.86 × 10^−1^ | 6.62 × 10^−1^ | 1.13	1.13 × 10^−2^ | 1.28 × 10^−2^ | 2.18 × 10^−2^	1.77 | 2.00 | 3.41
Scad and Related	1.91 × 10^−3^ | 2.42 × 10^−3^ | 4.12 × 10^−3^	3.50 × 10^−1^ | 4.43 × 10^−1^ | 7.54 × 10^−1^	4.38 × 10^−3^ | 5.54 × 10^−3^ | 9.43 × 10^−3^	8.01 × 10^−1^ | 1.01 | 1.73
Tuna	6.33 × 10^−4^ | 1.11 × 10^−3^ | 2.60 × 10^−3^	1.08 × 10^−1^ | 1.90 × 10^−1^ | 4.43 × 10^−1^	1.37 × 10^−3^ | 2.41 × 10^−3^ | 5.63 × 10^−3^	2.34 × 10^−1^ | 4.12 × 10^−1^ | 9.62 × 10^−1^
Fish-based snack	2.38 × 10^−4^ | 3.36 × 10^−4^ | 6.74 × 10^−4^	1.71 × 10^−1^ | 2.41 × 10^−1^ | 4.83 × 10^−1^	4.44 × 10^−4^ | 6.25 × 10^−4^ | 1.25 × 10^−3^	3.18 × 10^−1^ | 4.48 × 10^−1^ | 9.00 × 10^−1^
Fish Roe	9.62 × 10^−5^ | 1.06 × 10^−4^ | 1.31 × 10^−4^	9.68 × 10^−2^ | 1.07 × 10^−1^ | 1.32 × 10^−1^	1.14 × 10^−4^ | 1.26 × 10^−4^ | 1.56 × 10^−4^	1.15 × 10^−1^ | 1.27 × 10^−1^ | 1.57 × 10^−1^
Total	1.07 × 10^−1^ | 1.16 × 10^−1^ | 1.51 × 10^−1^	9.72 | 10.4 | 13.0	2.82 × 10^−1^ | 3.03 × 10^−1^ | 3.77 × 10^−1^	24.1 | 25.8 | 31.9

The hazard quotients (HQs) of each seafood category for both the general population and seafood consumers are presented at the mean ^a^ and 95th percentile (P95) intake levels ^b^. Seafood categories are listed in descending order according to the HQs LB mean intake of the general population ^c^.

## Data Availability

The original contributions presented in this study are included in the article/Supplementary Materials. Further inquiries can be directed to the corresponding author.

## Supplementary Materials

The following supporting information can be downloaded at: https://www.mdpi.com/article/10.3390/foods14234165/s1, Table S1: List of food samples tested with their respective cooking/preparation method and food category; Table S2: Kruskal–Wallis test using Chi-Square (df:3) distribution (right tailed) for the four PFAS occurrence means at 99% confidence level (α = 0.01); Table S3: One Way ANOVA test for the various cooking method at 95% confidence level (α = 0.05) and Tukey HSD.

## Abbreviations

The following abbreviations are used in this manuscript:

% HQ	Percentage contribution
ACN	Acetonitrile
ACS	American Chemical Society
ASEAN	Association of South East Asian Nations
ATSDR	Agency for Toxic Substances and Disease Registry
BfR	German Federal Institute for Risk Assessment
BK	Baking
BL	Boiling
BMDL10	Benchmark Dose Lower Confidence Limit 10%
BR	Braising
BW	Brewing
CE	Collision energy
CONTAM	EFSA Panel on Contaminants in the Food Chain
CUR	Curtain gas
DF	Deep frying
EFSA	European Food Safety Authority
EP	Eggs and egg products
EU	European Union
FAO	Food and Agriculture Organization of the United Nations
FASFC	Federal Agency for the Safety of the Food Chain
FS	Fish and seafood
FSANZ	Food Standards Australia and New Zealand
FSCJ	Food Safety Commission of Japan
GR	Grilling
GS1	Nebulizing gas
GS2	Drying gas
HB	Half-boiling
HBGVs	Health-based guidance values
HQs	Hazard quotients
kg bw	Kilograms per body weight
LB	Lower bound approach
LCMS	Liquid chromatography–mass spectrometry
LC-PFCAs	Long-chain perfluorocarboxylic acids
LOD	Limit of Detection
LOQ	Limit of Quantification
*m*/*z*	Mass-to-charge ratio
MB	Middle-bound approach
MeOH	Methanol
MgSO_4_	Magnesium sulphate
MP	Meat and meat products
MPFNA	^13^C_9_ perfluorononanoic acid
MPFOA	^13^C_8_ perfluorooctanoic acid
MPFOS	^13^C_8_ perfluorooctanoic sulfonate
MRM	Multiple reaction monitoring
NaCl	Anhydrous sodium chloride
NC	Uncooked
P95	95th percentile
PBPK	Pharmacokinetic modelling
PF	Pan frying
PFAS	Per- and poly-fluoroalkyl substances
PFDA	Perfluorodecanoic acid
PFHpA	Perfluoroheptanoic acid
PFHxS	Perfluorohexane sulfonate
PFNA	Perfluorononanoic acid
PFOA	Perfluorooctanoic acid
PFOS	Perfluorooctane sulfonate
POPRC	Stockholm Convention’s Persistent Organic Pollutants Review Committee
POPs	Persistent Organic Pollutants
PSA	Primary and secondary amines
Q1	Lower quartile, 25th percentile
Q3	Upper quartile, 75th percentile
IQR	Interquartile range
QuEChERS	Quick, Easy, Cheap, Effective, Rugged, and Safe
RC	Reconstitution
REACH	EU’s Registration, Evaluation, Authorisation and Restriction of Chemicals regulation
RS	Roasting
RSD	Relative standard deviation
SC	Sauces and condiments
SF	Stir frying
SM	Steaming
SP	Soup—liquid
SPE	Solid phase extraction
SS	Soup—solids
SVHC	Substance of Very High Concern under EU REACH
SW	Stewing
TDI	Tolerable daily intake
TDS	Total Diet Study
TWI	Tolerable weekly intake
UB	Upper bound approach
US FDA	United States Food and Drug Administration
WHO	World Health Organization

## Appendix A

**Table A1:** Spike recoveries of the PFAS for the various food types.

Food	PFOA	PFOS	PFNA	PFHxS
Dried mango	99%	95%	100%	85%
Chapati	100%	97%	98%	89%
Vegetable oil	99%	102%	96%	83%
Anchovy	98%	97%	98%	85%
Garlic	99%	103%	99%	84%
Tuna	94%	92%	98%	90%
Cucumber	102%	97%	101%	118%
Scallop	101%	98%	102%	115%
Spring onion	97%	93%	103%	92%
White jasmine rice	97%	93%	99%	97%
Pork, no fat	97%	93%	96%	94%
Peanut	95%	97%	97%	95%
Grape	87%	88%	100%	87%
Blueberry	91%	95%	98%	88%
Duck	90%	96%	100%	90%
White fish loin ^1^	101%	100%	107%	102%
Kang kong ^2^	102%	100%	105%	99%
Bread ^3^	100%	100%	103%	100%
Chicken ^1^	106%	97%	111%	104%
Spinach ^2^	103%	99%	105%	104%
Pastry ^3^	102%	96%	108%	112%
Pork loin ^1^	102%	99%	102%	101%
Bai Cai ^2^	102%	102%	101%	100%
Biscuit ^3^	100%	99%	100%	100%

Samples used for method validation of meat (^1^), vegetable (^2^), and carbohydrate (^3^).

**Table A2:** Percentage of detection of individual PFAS and summation of four PFAS (μg/kg) for the various types in the “fish and seafood” category. Arranged in decreasing order by the sum of the four PFAS.

“Fish and Seafood” Type	*n*	Rate of Detection				Sum of 4 PFAS (μg/kg)
		PFOA	PFOS	PFNA	PFHxS	
clam	2	100%	100%	100%	0%	3.17
crab	2	100%	100%	100%	0%	1.30
anchovy	10	30%	60%	70%	40%	0.87
fish roe	1	0%	100%	0%	0%	0.59
canned sardine	4	25%	75%	25%	0%	0.41
threadfin	1	0%	100%	100%	0%	0.31
catfish	1	0%	100%	0%	0%	0.23
scad and related	1	0%	100%	0%	0%	0.23
squid/cuttlefish	2	0%	100%	0%	0%	0.22
prawn/shrimp	8	0%	13%	13%	0%	0.18
mussel	4	50%	25%	0%	0%	0.18
fish-based snack	1	0%	100%	0%	0%	0.18
mackerel and related	1	0%	100%	0%	0%	0.17
cockle	6	50%	0%	33%	0%	0.16
canned tuna	1	0%	0%	0%	0%	0.13
salted fish and products	4	0%	25%	0%	0%	0.12
tuna	4	0%	50%	0%	0%	0.11
lobster/crayfish	2	0%	0%	0%	0%	0.10
prawn meatball	1	0%	0%	0%	0%	0.08
fish head, boiled	2	0%	0%	0%	0%	0.08
seabass	1	0%	0%	0%	0%	0.07
snapper	1	0%	0%	0%	0%	0.07
squid ball and related	1	0%	0%	0%	0%	0.06
scallop	10	0%	0%	0%	0%	0.03
fish nuggets and products	2	0%	0%	0%	0%	0.03
oyster	7	0%	0%	0%	0%	0.03
fish ball, cake, and related	1	0%	0%	0%	0%	0.03
grouper	1	0%	0%	0%	0%	0.03
salmon	1	0%	0%	0%	0%	0.02
trout/cod	8	0%	0%	0%	0%	0.01
sea cucumber	2	0%	0%	0%	0%	0.00

**Table A3:** Dietary exposure for the general population and consumers was calculated using dietary intake values from the survey, based on the mean and 95th percentile (P95) calculated using LB, MB, and UB of the sum of four PFAS compounds across quantifiable food categories.

	Total Dietary Exposure of PFAS (ng/kg bw/day), LB | MB | UB			
Food Category	Population		Consumers	
	Mean	P95	Mean	P95
Fish and seafood	2.7 × 10^−3^ | 3.6 × 10^−3^ | 6.1 × 10^−3^	1.6 × 10^−1^ | 2.0 × 10^−1^ | 3.3 × 10^−1^	1.0 × 10^−2^ | 1.4 × 10^−2^ | 2.3 × 10^−2^	4.0 × 10^−1^ | 5.4 × 10^−1^ | 9.2 × 10^−1^
Sauces and condiments	1.3 × 10^−4^ | 1.4 × 10^−3^ | 4.2 × 10^−3^	3.7 × 10^−3^ | 3.6 × 10^−2^ | 1.1 × 10^−1^	7.0 × 10^−4^ | 9.6 × 10^−3^ | 3.0 × 10^−2^	1.6 × 10^−2^ | 9.5 × 10^−2^ | 2.8 × 10^−1^
Eggs and egg products	3.1 × 10^−4^ | 4.6 × 10^−3^ | 1.5 × 10^−2^	1.3 × 10^−2^ | 7.7 × 10^−2^ | 2.4 × 10^−1^	7.7 × 10^−4^ | 2.8 × 10^−2^ | 9.1 × 10^−2^	3.3 × 10^−2^ | 1.9 × 10^−1^ | 5.6 × 10^−1^
Meat and meat products	1.3 × 10^−5^ | 3.7 × 10^−3^ | 1.2 × 10^−2^	2.3 × 10^−3^ | 1.0 × 10^−1^ | 3.4 × 10^−1^	8.3 × 10^−5^ | 1.8 × 10^−2^ | 6.0 × 10^−2^	4.5 × 10^−3^ | 2.5 × 10^−1^ | 8.3 × 10^−1^
Total	3.1 × 10^−3^ | 1.3 × 10^−2^ | 3.7 × 10^−2^	1.7 × 10^−1^ | 4.2 × 10^−1^ | 1.0	1.2 × 10^−2^ | 6.9 × 10^−2^ | 2.0 × 10^−1^	4.6 × 10^−1^ | 1.1 | 2.6

**Table A4:** The mean occurrence level and standard deviation (for types with 2 or more detections) were calculated using LB, MB, and UB for the various types in the “fish and seafood” category. Arranged in decreasing order of “Sum PFAS” values.

“Fish and Seafood” Type	Mean and Standard Deviation (μg/kg), LB | MB | UB				
	PFOA	PFOS	PFNA	PFHxS	Sum PFAS
clam	2.62 ± 0.27 | 2.62 ± 0.27 | 2.62 ± 0.27	0.24 ± 0.02 | 0.24 ± 0.02 | 0.24 ± 0.02	0.32 ± 0.00 | 0.32 ± 0.00 | 0.32 ± 0.00	0.05 ± 0.00 | 0.05 ± 0.00 | 0.10 ± 0.00	3.22 ± 0.30 | 3.22 ± 0.30 | 3.27 ± 0.30
crab	0.40 ± 0.15 | 0.40 ± 0.15 | 0.40 ± 0.15	0.60 ± 0.14 | 0.60 ± 0.14 | 0.60 ± 0.14	0.30 ± 0.09 | 0.30 ± 0.09 | 0.30 ± 0.09	0.00 ± 0.00 | 0.03 ± 0.00 | 0.10 ± 0.00	1.30 ± 0.37 | 1.33 ± 0.37 | 1.40 ± 0.37
anchovy	0.07 ± 0.05 | 0.08 ± 0.04 | 0.11 ± 0.02	0.52 ± 0.66 | 0.53 ± 0.65 | 0.56 ± 0.63	0.13 ± 0.10 | 0.14 ± 0.09 | 0.16 ± 0.06	0.13 ± 0.19 | 0.14 ± 0.18 | 0.17 ± 0.16	0.86 ± 0.92 | 0.90 ± 0.89 | 1.01 ± 0.82
fish roe	0.00 | 0.03 | 0.10	0.48 | 0.48 | 0.48	0.09 | 0.09 | 0.10	0.00 | 0.03 | 0.10	0.57 | 0.63 | 0.78
canned sardine	0.05 ± 0.06 | 0.06 ± 0.04 | 0.10 ± 0.01	0.31 ± 0.26 | 0.32 ± 0.25 | 0.33 ± 0.22	0.05 ± 0.05 | 0.06 ± 0.04 | 0.10 ± 0.01	0.00 ± 0.00 | 0.03 ± 0.00 | 0.10 ± 0.00	0.41 ± 0.36 | 0.47 ± 0.32 | 0.64 ± 0.24
threadfin	0.00 | 0.03 | 0.10	0.17 | 0.17 | 0.17	0.13 | 0.13 | 0.13	0.00 | 0.03 | 0.10	0.29 | 0.35 | 0.49
catfish	0.04 | 0.04 | 0.10	0.14 | 0.14 | 0.14	0.05 | 0.05 | 0.10	0.00 | 0.03 | 0.10	0.23 | 0.26 | 0.44
scad and related	0.00 | 0.03 | 0.10	0.19 | 0.19 | 0.19	0.04 | 0.04 | 0.10	0.00 | 0.03 | 0.10	0.23 | 0.29 | 0.49
squid/cuttlefish	0.03 ± 0.00 | 0.03 ± 0.00 | 0.10 ± 0.00	0.14 ± 0.02 | 0.14 ± 0.02 | 0.14 ± 0.02	0.05 ± 0.00 | 0.05 ± 0.00 | 0.10 ± 0.00	0.00 ± 0.00 | 0.03 ± 0.00 | 0.10 ± 0.00	0.22 ± 0.02 | 0.25 ± 0.02 | 0.44 ± 0.02
mussel	0.08 ± 0.03 | 0.08 ± 0.03 | 0.10 ± 0.00	0.07 ± 0.06 | 0.08 ± 0.05 | 0.11 ± 0.02	0.04 ± 0.03 | 0.04 ± 0.01 | 0.10 ± 0.00	0.00 ± 0.00 | 0.03 ± 0.00 | 0.10 ± 0.00	0.18 ± 0.09 | 0.23 ± 0.08 | 0.41 ± 0.02
prawn/shrimp	0.02 ± 0.02 | 0.03 ± 0.01 | 0.10 ± 0.00	0.08 ± 0.03 | 0.08 ± 0.02 | 0.10 ± 0.00	0.07 ± 0.03 | 0.07 ± 0.03 | 0.10 ± 0.00	0.00 ± 0.00 | 0.03 ± 0.00 | 0.10 ± 0.00	0.17 ± 0.08 | 0.22 ± 0.05 | 0.40 ± 0.00
mackerel and related	0.00 | 0.03 | 0.10	0.12 | 0.12 | 0.12	0.03 | 0.03 | 0.10	0.00 | 0.03 | 0.10	0.15 | 0.21 | 0.42
cockle	0.06 ± 0.07 | 0.08 ± 0.06 | 0.11 ± 0.02	0.01 ± 0.01 | 0.03 ± 0.00 | 0.10 ± 0.00	0.08 ± 0.03 | 0.08 ± 0.03 | 0.10 ± 0.01	0.00 ± 0.00 | 0.03 ± 0.00 | 0.10 ± 0.00	0.15 ± 0.11 | 0.22 ± 0.09 | 0.42 ± 0.03
fish-based snack	0.00 | 0.03 | 0.10	0.12 | 0.12 | 0.12	0.03 | 0.03 | 0.10	0.00 | 0.03 | 0.10	0.15 | 0.21 | 0.42
canned tuna	0.00 | 0.03 | 0.10	0.08 | 0.08 | 0.10	0.03 | 0.03 | 0.10	0.00 | 0.03 | 0.10	0.11 | 0.17 | 0.40
salted fish and products	0.00 ± 0.00 | 0.03 ± 0.00 | 0.10 ± 0.00	0.08 ± 0.03 | 0.08 ± 0.03 | 0.10 ± 0.00	0.04 ± 0.02 | 0.04 ± 0.01 | 0.10 ± 0.00	0.00 ± 0.00 | 0.03 ± 0.00 | 0.10 ± 0.00	0.11 ± 0.04 | 0.18 ± 0.03 | 0.40 ± 0.00
tuna	0.00 ± 0.00 | 0.03 ± 0.00 | 0.10 ± 0.00	0.08 ± 0.04 | 0.08 ± 0.04 | 0.11 ± 0.01	0.02 ± 0.02 | 0.03 ± 0.00 | 0.10 ± 0.00	0.00 ± 0.00 | 0.03 ± 0.00 | 0.10 ± 0.00	0.10 ± 0.06 | 0.17 ± 0.04 | 0.41 ± 0.01
lobster/crayfish	0.04 ± 0.00 | 0.04 ± 0.00 | 0.10 ± 0.00	0.00 ± 0.00 | 0.03 ± 0.00 | 0.10 ± 0.00	0.04 ± 0.01 | 0.04 ± 0.01 | 0.10 ± 0.00	0.00 ± 0.00 | 0.03 ± 0.00 | 0.10 ± 0.00	0.08 ± 0.01 | 0.14 ± 0.01 | 0.40 ± 0.00
seabass	0.00 | 0.03 | 0.10	0.06 | 0.06 | 0.10	0.00 | 0.03 | 0.10	0.00 | 0.03 | 0.10	0.06 | 0.15 | 0.40
fish head	0.00 ± 0.00 | 0.03 ± 0.00 | 0.10 ± 0.00	0.06 ± 0.00 | 0.06 ± 0.00 | 0.10 ± 0.00	0.00 ± 0.00 | 0.03 ± 0.00 | 0.10 ± 0.00	0.00 ± 0.00 | 0.03 ± 0.00 | 0.10 ± 0.00	0.06 ± 0.00 | 0.15 ± 0.00 | 0.40 ± 0.00
snapper	0.00 | 0.03 | 0.10	0.06 | 0.06 | 0.10	0.00 | 0.03 | 0.10	0.00 | 0.03 | 0.10	0.06 | 0.15 | 0.40
prawn meatball	0.00 | 0.03 | 0.10	0.05 | 0.05 | 0.10	0.00 | 0.03 | 0.10	0.00 | 0.03 | 0.10	0.05 | 0.14 | 0.40
squid ball and related	0.00 | 0.03 | 0.10	0.04 | 0.04 | 0.10	0.00 | 0.03 | 0.10	0.00 | 0.03 | 0.10	0.04 | 0.13 | 0.40
scallop	0.02 ± 0.03 | 0.04 ± 0.01 | 0.10 ± 0.00	0.00 ± 0.00 | 0.03 ± 0.00 | 0.10 ± 0.00	0.00 ± 0.00 | 0.03 ± 0.00 | 0.10 ± 0.00	0.00 ± 0.00 | 0.03 ± 0.00 | 0.10 ± 0.00	0.02 ± 0.03 | 0.13 ± 0.01 | 0.40 ± 0.00
oyster	0.01 ± 0.02 | 0.03 ± 0.00 | 0.10 ± 0.00	0.00 ± 0.00 | 0.03 ± 0.00 | 0.10 ± 0.00	0.00 ± 0.00 | 0.03 ± 0.00 | 0.10 ± 0.00	0.00 ± 0.00 | 0.03 ± 0.00 | 0.10 ± 0.00	0.01 ± 0.02 | 0.12 ± 0.00 | 0.40 ± 0.00
fish ball, cake, and related	0.00 | 0.03 | 0.10	0.00 | 0.03 | 0.10	0.00 | 0.03 | 0.10	0.00 | 0.03 | 0.10	0.00 | 0.12 | 0.40
fish nuggets and products	0.00 ± 0.00 | 0.03 ± 0.00 | 0.10 ± 0.00	0.00 ± 0.00 | 0.03 ± 0.00 | 0.10 ± 0.00	0.00 ± 0.00 | 0.03 ± 0.00 | 0.10 ± 0.00	0.00 ± 0.00 | 0.03 ± 0.00 | 0.10 ± 0.00	0.00 ± 0.00 | 0.12 ± 0.00 | 0.40 ± 0.00
grouper	0.00 | 0.03 | 0.10	0.00 | 0.03 | 0.10	0.00 | 0.03 | 0.10	0.00 | 0.03 | 0.10	0.00 | 0.12 | 0.40
salmon	0.00 | 0.03 | 0.10	0.00 | 0.03 | 0.10	0.00 | 0.03 | 0.10	0.00 | 0.03 | 0.10	0.00 | 0.12 | 0.40
sea cucumber	0.00 ± 0.00 | 0.03 ± 0.00 | 0.10 ± 0.00	0.00 ± 0.00 | 0.03 ± 0.00 | 0.10 ± 0.00	0.00 ± 0.00 | 0.03 ± 0.00 | 0.10 ± 0.00	0.00 ± 0.00 | 0.03 ± 0.00 | 0.10 ± 0.00	0.00 ± 0.00 | 0.12 ± 0.00 | 0.4 ± 0.00
trout/cod	0.00 ± 0.00 | 0.03 ± 0.00 | 0.10 ± 0.00	0.00 ± 0.00 | 0.03 ± 0.00 | 0.10 ± 0.00	0.00 ± 0.00 | 0.03 ± 0.00 | 0.10 ± 0.00	0.00 ± 0.00 | 0.03 ± 0.00 | 0.10 ± 0.00	0.00 ± 0.00 | 0.12 ± 0.00 | 0.40 ± 0.00

**Table A5:** Mean and 95th percentile (P95) dietary exposure of sum of four PFAS from the various seafood categories calculated using LB, MB, and UB for the whole population and for consumers only, arranged in descending order for LB population mean.

	Dietary Exposure of Sum of Four PFAS (ng/kg bw/day), LB | MB | UB			
	Population		Consumers	
Seafood Category	Mean	P95	Mean	P95
Clam	2.46 × 10^−2^ | 2.46 × 10^−2^ | 2.50 × 10^−2^	6.54 × 10^−2^ | 6.54 × 10^−2^ | 6.65 × 10^−2^	2.99 | 2.99 | 3.04	7.97 | 7.97 | 8.10
Crab	1.50 × 10^−2^ | 1.54 × 10^−2^ | 1.62 × 10^−2^	3.14 × 10^−2^ | 3.21 × 10^−2^ | 3.38 × 10^−2^	1.21 | 1.24 | 1.30	2.53 | 2.58 | 2.72
Mackerel and Related	7.03 × 10^−3^ | 9.86 × 10^−3^ | 1.98 × 10^−2^	1.39 × 10^−2^ | 1.95 × 10^−2^ | 3.90 × 10^−2^	2.12 × 10^−1^ | 2.98 × 10^−1^ | 5.97 × 10^−1^	4.19 × 10^−1^ | 5.88 × 10^−1^ | 1.18
Anchovy	6.87 × 10^−3^ | 7.19 × 10^−3^ | 8.04 × 10^−3^	4.02 × 10^−2^ | 4.20 × 10^−2^ | 4.70 × 10^−2^	9.23 × 10^−2^ | 9.65 × 10^−2^ | 1.08 × 10^−1^	2.55 × 10^−1^ | 2.67 × 10^−1^ | 2.98 × 10^−1^
Squid/Cuttlefish	4.22 × 10^−3^ | 4.80 × 10^−3^ | 8.50 × 10^−3^	3.69 × 10^−3^ | 4.20 × 10^−3^ | 7.43 × 10^−3^	9.45 × 10^−2^ | 1.08 × 10^−1^ | 1.90 × 10^−1^	2.88 × 10^−1^ | 3.28 × 10^−1^ | 5.80 × 10^−1^
Threadfin	2.68 × 10^−3^ | 3.23 × 10^−3^ | 4.52 × 10^−3^	4.31 × 10^−3^ | 5.19 × 10^−3^ | 7.27 × 10^−3^	2.57 × 10^−1^ | 3.10 × 10^−1^ | 4.34 × 10^−1^	4.13 × 10^−1^ | 4.98 × 10^−1^ | 6.97 × 10^−1^
Catfish	2.36 × 10^−3^ | 2.67 × 10^−3^ | 4.54 × 10^−3^	7.13 × 10^−3^ | 8.06 × 10^−3^ | 1.37 × 10^−2^	3.69 × 10^−1^ | 4.17 × 10^−1^ | 7.10 × 10^−1^	1.12 | 1.26 | 2.15
Canned sardine	1.27 × 10^−3^ | 1.95 × 10^−3^ | 4.53 × 10^−3^	2.36 × 10^−3^ | 3.62 × 10^−3^ | 8.42 × 10^−3^	7.65 × 10^−2^ | 1.17 × 10^−1^ | 2.73 × 10^−1^	1.42 × 10^−1^ | 2.19 × 10^−1^ | 5.09 × 10^−1^
Scad and Related	1.20 × 10^−3^ | 1.52 × 10^−3^ | 2.60 × 10^−3^	2.76 × 10^−3^ | 3.49 × 10^−3^ | 5.94 × 10^−3^	2.21 × 10^−1^ | 2.79 × 10^−1^ | 4.75 × 10^−1^	5.05 × 10^−1^ | 6.39 × 10^−1^ | 1.09
Tuna	3.99 × 10^−4^ | 7.00 × 10^−4^ | 1.64 × 10^−3^	8.65 × 10^−4^ | 1.52 × 10^−3^ | 3.55 × 10^−3^	6.81 × 10^−2^ | 1.20 × 10^−1^ | 2.79 × 10^−1^	1.48 × 10^−1^ | 2.59 × 10^−1^ | 6.06 × 10^−1^
Fish-based snack	1.50 × 10^−4^ | 2.11 × 10^−4^ | 4.24 × 10^−4^	2.80 × 10^−4^ | 3.94 × 10^−4^ | 7.90 × 10^−4^	1.08 × 10^−1^ | 1.52 × 10^−1^ | 3.04 × 10^−1^	2.01 × 10^−1^ | 2.82 × 10^−1^ | 5.67 × 10^−1^
Fish Roe	6.06 × 10^−5^ | 6.69 × 10^−5^ | 8.28 × 10^−5^	7.18 × 10^−5^ | 7.93 × 10^−5^ | 9.81 × 10^−5^	6.10 × 10^−2^ | 6.74 × 10^−2^ | 8.33 × 10^−2^	7.23 × 10^−2^ | 7.99 × 10^−2^ | 9.88 × 10^−2^

**Table A6:** Comparison of PFAS mean LB values (μg/kg) for the various cooking methods for food types that have detected values, arranged in increasing order of cooking intensity. Values below LOQ were reported as zero. Sample sizes are indicated under “*n*”.

Food	Cooking Method	*n*	PFOA	PFOS	PFNA	PFHxS	4 PFAS
Anchovy	Soup liquid	3	0.00	0.00	0.00	0.00	0.00
	Soup solid	3	0.00	0.43	0.15	0.00	0.58
	Pan fry	3	0.00	0.70	0.18	0.14	1.02
	Deep fry	1	0.11	0.54	0.20	0.13	0.98
Mussel	Steam	1	0.10	0.00	0.00	0.00	0.10
	Stir fry	3	0.10	0.14	0.00	0.00	0.24
Squid and cuttlefish	Boil	2	0.00	0.15	0.00	0.00	0.15
	Stir fry	1	0.00	0.12	0.00	0.00	0.12
Cockle	Uncooked (Raw)	2	0.10	0.00	0.00	0.00	0.10
	Boil	2	0.14	0.00	0.11	0.00	0.24
	Stir fry	2	0.15	0.00	0.12	0.00	0.27
Crab	Boil	1	0.30	0.50	0.24	0.00	1.04
	Stir fry	1	0.51	0.70	0.36	0.00	1.56
Clam	Boil	1	2.43	0.22	0.31	0.00	2.96
	Stir fry	1	2.81	0.25	0.32	0.00	3.38
