# Supplementary material for: Occurrence and Dietary Exposure of PFAS in Singapore: Insights from a Total Diet Study
Source: Foods. 2025 Dec 4;14(23):4165. doi: 10.3390/foods14234165 (PMC12691853; doi:10.3390/foods14234165)
Supplement: Supplementary file 1 [file foods-14-04165-s001.zip › foods-3939776-supplementary.pdf]

Supporting Information

Occurrence and Dietary Exposure of PFAS in Singapore: In-sights from a Total Diet Study

Ignatius Lim [1], Ping Shen [1], *, Wei Min Ang [1], Chin Yee Soon [1], Raymond Rong Sheng Shi [1], Wesley Zongrong Yu [1], and Sheot Harn Chan [1,2]

1 National Centre for Food Science, Singapore Food Agency, 7 International Business Park, Singapore 609919, Singapore

2 Faculty of Science, Department of Food Science & Technology, National University of Singapore, Science Drive 2, Singapore 117542, Singapore

** Correspondence: ping_shen@sfa.gov.sg*

Table S1: List of food sample tested with their respective cooking / preparation method and food category.

Food sample	Cooking / Preparation method	Food Category
Biscuit	No cooking	Bakery products
Bun, custard	No cooking	Bakery products
Cake	No cooking	Bakery products
Chinese fried dough (You tiao)	Deep fry	Bakery products
Chinese fried dough (You tiao)	Deep fry	Bakery products
Kueh, no coconut	No cooking	Bakery products
Kueh, with coconut	No cooking	Bakery products
Pancake	No cooking	Bakery products
Pastry	No cooking	Bakery products
Puff and pie	No cooking	Bakery products
Savoury bun (bao)	No cooking	Bakery products
Sweet bun (bao)	No cooking	Bakery products
White bread	No cooking	Bakery products
Wholemeal bread	No cooking	Bakery products
Alcohol, beer	Ready to eat	Beverages (including bottled water)
Alcohol, red wine	No cooking	Beverages (including bottled water)
Alcohol, white wine	No cooking	Beverages (including bottled water)
Boba (tapioca pearl)	No cooking	Beverages (including bottled water)
Bottled water	No cooking	Beverages (including bottled water)
Carbonated soft drink	No cooking	Beverages (including bottled water)
Cocoa powder	Reconstitute	Beverages (including bottled water)
Energy drink	No cooking	Beverages (including bottled water)
Freshly brewed coffee	Brew	Beverages (including bottled water)
Green tea and chinese tea	Brew	Beverages (including bottled water)
Instant coffee	Reconstitute	Beverages (including bottled water)
Isotonic sport drink	No cooking	Beverages (including bottled water)
Juice drink (not freshly prepared)	No cooking	Beverages (including bottled water)

Malted, cereal or chocolate drink	Reconstitute	Beverages (including bottled water)
Milk tea	No cooking	Beverages (including bottled water)
Msg	No cooking	Beverages (including bottled water)
Non-CO2 sweetened beverage	No cooking	Beverages (including bottled water)
Red (english) tea	Brew	Beverages (including bottled water)
Salt	No cooking	Beverages (including bottled water)
Sugar, white	No cooking	Beverages (including bottled water)
Sugarcane juice	No cooking	Beverages (including bottled water)
Broccoli	Boil	Brassica vegetables
Broccoli	Boil	Brassica vegetables
Broccoli	Stir fry	Brassica vegetables
Broccoli	Stir fry	Brassica vegetables
Carrot cake	Pan fry	Brassica vegetables
Cauliflower	Boil	Brassica vegetables
Cauliflower	Stir fry	Brassica vegetables
Preserved / pickled vegetable	Boil	Brassica vegetables
Rice flour noodle dish with coconut and palm sugar (Putu mayam)	Steam	Brassica vegetables
Curry gravy (fish)	Boil	Composite foods
Curry gravy (meat)	Boil	Composite foods
Dhal	Boil	Composite foods
Meat dumpling	Ready to eat	Composite foods
Mushroom soup (canned, instant)	Boil	Composite foods
Pizza	No cooking	Composite foods
Candy / sweet	No cooking	Confectionary
Chocolate	No cooking	Confectionary
Dairy pudding	No cooking	Confectionary
Honey	No cooking	Confectionary
Jelly	No cooking	Confectionary
Syrup	No cooking	Confectionary

Century egg	Boil	Eggs and egg products
Century egg	No cooking	Eggs and egg products
Century egg	Ready to eat	Eggs and egg products
Egg	Boil	Eggs and egg products
Egg	Braised	Eggs and egg products
Egg	Half-boil	Eggs and egg products
Egg	Pan fry	Eggs and egg products
Egg	Steam	Eggs and egg products
Egg tofu	Boil	Eggs and egg products
Egg tofu	Pan fry	Eggs and egg products
Fermented soybean product	Stir fry	Eggs and egg products
Salted duck egg	Boil	Eggs and egg products
Soy milk	No cooking	Eggs and egg products
Soybean	Soup (liquid)	Eggs and egg products
Soybean	Soup (solids)	Eggs and egg products
Animal fat	Deep fried	Fats and oils
Animal fat	Deep fry	Fats and oils
Oil, sesame	No cooking	Fats and oils
Olive oil	No cooking	Fats and oils
Vegetable oil	Deep fry	Fats and oils
Vegetable oil	No cooking	Fats and oils
Vegetable oil	Stir fry	Fats and oils
Anchovy	Deep fry	Fish and seafood
Anchovy	Pan fry	Fish and seafood
Anchovy	Pan fry	Fish and seafood
Anchovy	Pan fry	Fish and seafood
Anchovy	Soup (liquid)	Fish and seafood
Anchovy	Soup (liquid)	Fish and seafood
Anchovy	Soup (liquid)	Fish and seafood
Anchovy	Soup (solids)	Fish and seafood
Anchovy	Soup (solids)	Fish and seafood

Anchovy	Soup (solids)	Fish and seafood
Canned sardine	Stew	Fish and seafood
Canned sardine	Stew	Fish and seafood
Canned sardine	Stir fry	Fish and seafood
Canned sardine	Stir fry	Fish and seafood
Canned tuna	No cooking	Fish and seafood
Catfish	Deep fry	Fish and seafood
Clam	Boil	Fish and seafood
Clam	Stir fry	Fish and seafood
Cockle	Boil	Fish and seafood
Cockle	Boil	Fish and seafood
Cockle	Ready to eat	Fish and seafood
Cockle	Ready to eat	Fish and seafood
Cockle	Stir fry	Fish and seafood
Cockle	Stir fry	Fish and seafood
Crab	Boil	Fish and seafood
Crab	Stir fry	Fish and seafood
Fish ball, fish cake & related	Boil	Fish and seafood
Fish based snack	No cooking	Fish and seafood
Fish Head, boiled	Soup (liquid)	Fish and seafood
Fish Head, boiled	Soup (solids)	Fish and seafood
Fish nugget and related product	Bake	Fish and seafood
Fish nugget and related product	Deep fry	Fish and seafood
Fish roe	Ready to eat	Fish and seafood
Grouper	Steam	Fish and seafood
Kuning and related fishes	Deep fry	Fish and seafood
Lobster / crayfish	Boil	Fish and seafood
Lobster / crayfish	Stir fry	Fish and seafood
Mackerel & fishes (e.g. Batang)	Pan fry	Fish and seafood
Mussel	Steam	Fish and seafood
Mussel	Steam	Fish and seafood

Mussel	Stir fry	Fish and seafood
Mussel	Stir fry	Fish and seafood
Oyster	Boil	Fish and seafood
Oyster	Boil	Fish and seafood
Oyster	Ready to eat	Fish and seafood
Oyster	Ready to eat	Fish and seafood
Oyster	Stir fry	Fish and seafood
Oyster	Stir fry	Fish and seafood
Oyster Omelette (Orh luak)	Pan fry	Fish and seafood
Prawn / shrimp	Boil	Fish and seafood
Prawn / shrimp	Boil	Fish and seafood
Prawn / shrimp	Deep fry	Fish and seafood
Prawn / shrimp	Deep fry	Fish and seafood
Prawn / shrimp	Soup (liquid)	Fish and seafood
Prawn / shrimp	Soup (solids)	Fish and seafood
Prawn / shrimp	Stir fry	Fish and seafood
Prawn / shrimp	Stir fry	Fish and seafood
Prawn meat ball	Boil	Fish and seafood
Salmon	Pan fry	Fish and seafood
Salted fish and related product	Deep fry	Fish and seafood
Salted fish and related product	Deep fry	Fish and seafood
Salted fish and related product	Stir fry	Fish and seafood
Salted fish and related product	Stir fry	Fish and seafood
Scallop	Boil	Fish and seafood
Scallop	Boil	Fish and seafood
Scallop	Ready to eat	Fish and seafood
Scallop	Ready to eat	Fish and seafood
Scallop	Soup (liquid)	Fish and seafood
Scallop	Soup (liquid)	Fish and seafood
Scallop	Soup (solids)	Fish and seafood
Scallop	Soup (solids)	Fish and seafood

Scallop	Stir fry	Fish and seafood
Scallop	Stir fry	Fish and seafood
Sea cucumber	Boil	Fish and seafood
Sea cucumber	Boil	Fish and seafood
Seabass	Steam	Fish and seafood
Snapper	Steam	Fish and seafood
Squid / cuttlefish	Boil	Fish and seafood
Squid / cuttlefish	Stir fry	Fish and seafood
Squid ball and related product	Boil	Fish and seafood
Threadfin (ngor he)	Steam	Fish and seafood
Trout / cod	Boil	Fish and seafood
Trout / cod	Boil	Fish and seafood
Trout / cod	Deep fry	Fish and seafood
Trout / cod	Deep fry	Fish and seafood
Trout / cod	Steam	Fish and seafood
Trout / cod	Steam	Fish and seafood
Trout / cod	Stew	Fish and seafood
Trout / cod	Stew	Fish and seafood
Tuna	Braised	Fish and seafood
Tuna	Braised	Fish and seafood
Tuna	Pan fry	Fish and seafood
Tuna	Pan fry	Fish and seafood
Aloe vera	Ready to eat	Fruit and fruit products
Apple	No cooking	Fruit and fruit products
Apple	No cooking	Fruit and fruit products
Avocado	No cooking	Fruit and fruit products
Avocado	Ready to eat	Fruit and fruit products
Banana	No cooking	Fruit and fruit products
Blueberry	No cooking	Fruit and fruit products
Blueberry	No cooking	Fruit and fruit products
Coconut flesh	Ready to eat	Fruit and fruit products

Coconut milk	No cooking	Fruit and fruit products
Coconut water	Ready to eat	Fruit and fruit products
Dragonfruit	No cooking	Fruit and fruit products
Dragonfruit	Ready to eat	Fruit and fruit products
Dried apricot	No cooking	Fruit and fruit products
Dried dates	No cooking	Fruit and fruit products
Dried mango	No cooking	Fruit and fruit products
Dried prunes	No cooking	Fruit and fruit products
Dried raisins	No cooking	Fruit and fruit products
Durian	No cooking	Fruit and fruit products
Durian	No cooking	Fruit and fruit products
Grape	No cooking	Fruit and fruit products
Grape	No cooking	Fruit and fruit products
Guava	No cooking	Fruit and fruit products
Jackfruit	Ready to eat	Fruit and fruit products
Jam	No cooking	Fruit and fruit products
Kaya	No cooking	Fruit and fruit products
Kiwi	No cooking	Fruit and fruit products
Kiwi	Ready to eat	Fruit and fruit products
Lime / calamansi	Ready to eat	Fruit and fruit products
Longan	Ready to eat	Fruit and fruit products
Mandarin orange	No cooking	Fruit and fruit products
Mango	No cooking	Fruit and fruit products
Mango	Ready to eat	Fruit and fruit products
Mangosteen	Ready to eat	Fruit and fruit products
Melon	Ready to eat	Fruit and fruit products
Orange	No cooking	Fruit and fruit products
Orange	Ready to eat	Fruit and fruit products
Papaya	Ready to eat	Fruit and fruit products
Peach	No cooking	Fruit and fruit products
Peach	Ready to eat	Fruit and fruit products

Pear	No cooking	Fruit and fruit products
Pear	Ready to eat	Fruit and fruit products
Persimmon	No cooking	Fruit and fruit products
Persimmon	Ready to eat	Fruit and fruit products
Pineapple	Ready to eat	Fruit and fruit products
Plum	No cooking	Fruit and fruit products
Plum	No cooking	Fruit and fruit products
Pomelo	No cooking	Fruit and fruit products
Pomelo	Ready to eat	Fruit and fruit products
Rambutan	Ready to eat	Fruit and fruit products
Soursop	Ready to eat	Fruit and fruit products
Starfruit	No cooking	Fruit and fruit products
Starfruit	No cooking	Fruit and fruit products
Strawberry	No cooking	Fruit and fruit products
Strawberry	No cooking	Fruit and fruit products
Water chestnut	Ready to eat	Fruit and fruit products
Watermelon	Ready to eat	Fruit and fruit products
Bitter gourd	Boil	Fruiting vegetables
Bitter gourd	Stir fry	Fruiting vegetables
Bottle gourd	Stir fry	Fruiting vegetables
Brinjal	Boil	Fruiting vegetables
Brinjal	Steam	Fruiting vegetables
Brinjal	Stir fry	Fruiting vegetables
Capsicum	Boil	Fruiting vegetables
Capsicum	Stir fry	Fruiting vegetables
Chilli	Boil	Fruiting vegetables
Chilli	Ready to eat	Fruiting vegetables
Chilli	Stir fry	Fruiting vegetables
Cucumber	Ready to eat	Fruiting vegetables
Ladies finger	Boil	Fruiting vegetables
Ladies finger	Boil	Fruiting vegetables

Ladies finger	Stir fry	Fruiting vegetables
Ladies finger	Stir fry	Fruiting vegetables
Tomato	Boil	Fruiting vegetables
Tomato	Ready to eat	Fruiting vegetables
Tomato	Stir fry	Fruiting vegetables
Winter melon	Boil	Fruiting vegetables
Zucchini	Stir fry	Fruiting vegetables
Black jelly fungus	Boil	Fungi, seaweed
Canned mushroom	Boil	Fungi, seaweed
Corn	Boil	Fungi, seaweed
Dried mushroom	Boil	Fungi, seaweed
Dried seaweed	No cooking	Fungi, seaweed
Fresh mushroom	Boil	Fungi, seaweed
Fresh mushroom	Stir fry	Fungi, seaweed
Kale	Boil	Fungi, seaweed
Kale	Stir fry	Fungi, seaweed
Kimchi	No cooking	Fungi, seaweed
Seaweed	Boil	Fungi, seaweed
Seaweed	Soup (liquid)	Fungi, seaweed
Seaweed	Soup (solids)	Fungi, seaweed
White jelly fungus	Boil	Fungi, seaweed
White jelly fungus	Boil	Fungi, seaweed
Barley	Soup (liquid)	Grains and grain-based products
Barley	Soup (liquid)	Grains and grain-based products
Barley	Soup (solids)	Grains and grain-based products
Barley	Soup (solids)	Grains and grain-based products
Breakfast cereal	Ready to eat	Grains and grain-based products
Brown rice	Steam	Grains and grain-based products
Brown rice	Stir fry	Grains and grain-based products
Buckwheat noodle	Boil	Grains and grain-based products
Chapati	No cooking	Grains and grain-based products

Coconut rice	Steam	Grains and grain-based products
Glutinous rice	Steam	Grains and grain-based products
Idli	No cooking	Grains and grain-based products
Instant noodle	Boil	Grains and grain-based products
Noodles, wheat (e.g. Ban mian)	Boil	Grains and grain-based products
Oat	Boil	Grains and grain-based products
Pasta	Boil	Grains and grain-based products
Rice dumpling	No cooking	Grains and grain-based products
Rice dumpling	No cooking	Grains and grain-based products
Rice noodle	Boil	Grains and grain-based products
Rice noodle	Boil	Grains and grain-based products
Rice noodle	Stir fry	Grains and grain-based products
Roti prata	No cooking	Grains and grain-based products
Thosai	No cooking	Grains and grain-based products
Udon	Boil	Grains and grain-based products
Vermicelli	Boil	Grains and grain-based products
Vermicelli	Boil	Grains and grain-based products
White basmati rice	Boil	Grains and grain-based products
White basmati rice	Boil	Grains and grain-based products
White basmati rice	Steam	Grains and grain-based products
White japonica rice	Steam	Grains and grain-based products
White jasmine rice	Steam	Grains and grain-based products
White jasmine rice	Stir fry	Grains and grain-based products
Yellow and egg noodle	Boil	Grains and grain-based products
Infant cereal	Reconstitute	Infant food products
Infant formulae (1 to 9 yrs)	Reconstitute	Infant food products
Milk, condensed	No cooking	Infant food products
Milk, low fat	No cooking	Infant food products
Milk, powdered, low fat	Reconstitute	Infant food products
Milk, powdered, regular	Reconstitute	Infant food products
Milk, powdered, skimmed	Reconstitute	Infant food products

Milk, regular	No cooking	Infant food products
Milk, skimmed	No cooking	Infant food products
Soybean formulae (0 to 12 mths)	Reconstitute	Infant food products
Teething rusk	No cooking	Infant food products
Bai cai	Boil	Leafy vegetables, herbs
Bai cai	Stir fry	Leafy vegetables, herbs
Basil	Stir fry	Leafy vegetables, herbs
Bayam	Boil	Leafy vegetables, herbs
Bayam	Stir fry	Leafy vegetables, herbs
Cabbage	Boil	Leafy vegetables, herbs
Cabbage	Stir fry	Leafy vegetables, herbs
Chinese celery	Boil	Leafy vegetables, herbs
Chinese celery	Stir fry	Leafy vegetables, herbs
Chye sim	Boil	Leafy vegetables, herbs
Chye sim	Stir fry	Leafy vegetables, herbs
Coriander	Boil	Leafy vegetables, herbs
Coriander	Ready to eat	Leafy vegetables, herbs
Coriander	Stir fry	Leafy vegetables, herbs
Kailan	Boil	Leafy vegetables, herbs
Kailan	Stir fry	Leafy vegetables, herbs
Kang kong	Boil	Leafy vegetables, herbs
Kang kong	Stir fry	Leafy vegetables, herbs
Lettuce	Boil	Leafy vegetables, herbs
Lettuce	Ready to eat	Leafy vegetables, herbs
Lettuce	Stir fry	Leafy vegetables, herbs
Malabar	Boil	Leafy vegetables, herbs
Malabar	Stir fry	Leafy vegetables, herbs
Mint leaf	Ready to eat	Leafy vegetables, herbs
Nai bai	Boil	Leafy vegetables, herbs
Nai bai	Stir fry	Leafy vegetables, herbs
Pandan leaf	Soup (liquid)	Leafy vegetables, herbs

Pandan leaf	Soup (solids)	Leafy vegetables, herbs
Spinach	Boil	Leafy vegetables, herbs
Spinach	Stir fry	Leafy vegetables, herbs
Sweet potato leaf	Boil	Leafy vegetables, herbs
Sweet potato leaf	Stir fry	Leafy vegetables, herbs
Watercress	Boil	Leafy vegetables, herbs
Xiao bai cai	Boil	Leafy vegetables, herbs
Xiao bai cai	Stir fry	Leafy vegetables, herbs
Beancurd dessert	No cooking	Legumes, Nuts & Seeds
Beancurd, tofu, tau kwa, taupok	Pan fry	Legumes, Nuts & Seeds
Beansprout	Boil	Legumes, Nuts & Seeds
Beansprout	Stir fry	Legumes, Nuts & Seeds
Dried bean	Boil	Legumes, Nuts & Seeds
Lentil	Boil	Legumes, Nuts & Seeds
Lentil	Stir fry	Legumes, Nuts & Seeds
Long bean	Stir fry	Legumes, Nuts & Seeds
Pea	Boil	Legumes, Nuts & Seeds
Pea	Stir fry	Legumes, Nuts & Seeds
Pea sprout	Boil	Legumes, Nuts & Seeds
Pea sprout	Stir fry	Legumes, Nuts & Seeds
Peanut	Deep fry	Legumes, Nuts & Seeds
Peanut butter	No cooking	Legumes, Nuts & Seeds
Sesame seed	No cooking	Legumes, Nuts & Seeds
Soy curd, tau kwa, taupok, tempeh	Boil	Legumes, Nuts & Seeds
Soy curd, tau kwa, taupok, tempeh	Pan fry	Legumes, Nuts & Seeds
Beef bacon	Pan fry	Meat and meat products
Beef, no fat	Boil	Meat and meat products
Beef, no fat	Boil	Meat and meat products
Beef, no fat	Grill	Meat and meat products

Beef, no fat	Grill	Meat and meat products
Beef, no fat	Pan fry	Meat and meat products
Beef, no fat	Stew	Meat and meat products
Beef, no fat	Stew	Meat and meat products
Beef, no fat	Stir fry	Meat and meat products
Beef, no fat	Stir fry	Meat and meat products
Beef, with fat	Boil	Meat and meat products
Beef, with fat	Boil	Meat and meat products
Beef, with fat	Grill	Meat and meat products
Beef, with fat	Grill	Meat and meat products
Beef, with fat	Pan fry	Meat and meat products
Beef, with fat	Stew	Meat and meat products
Beef, with fat	Stew	Meat and meat products
Beef, with fat	Stir fry	Meat and meat products
Beef, with fat	Stir fry	Meat and meat products
Chicken	Boil	Meat and meat products
Chicken	Deep fry	Meat and meat products
Chicken	Roast	Meat and meat products
Chicken	Soup (liquid)	Meat and meat products
Chicken	Soup (solids)	Meat and meat products
Chicken	Steam	Meat and meat products
Chicken	Stir fry	Meat and meat products
Chicken ham, bologna	Ready to eat	Meat and meat products
Chicken nugget	Bake	Meat and meat products
Chicken nugget	Deep fry	Meat and meat products
Chicken sausage, hot dog, cocktail	Boil	Meat and meat products
Chicken sausage, hot dog, cocktail	Pan fry	Meat and meat products
Chicken sausage, hot dog, cocktail	Pan fry	Meat and meat products
Chinese sausage (eg. Lup cheong)	Stir fry	Meat and meat products
Duck	Boil	Meat and meat products
Duck	Braised	Meat and meat products

Duck	Roast	Meat and meat products
Mutton, no fat	Boil	Meat and meat products
Mutton, no fat	Stew	Meat and meat products
Mutton, no fat	Stir fry	Meat and meat products
Pork bacon	Pan fry	Meat and meat products
Pork ham, bologna	Ready to eat	Meat and meat products
Pork luncheon meat	Deep fry	Meat and meat products
Pork luncheon meat	No cooking	Meat and meat products
Pork luncheon meat	Pan fry	Meat and meat products
Pork luncheon meat	Ready to eat	Meat and meat products
Pork meat ball	Boil	Meat and meat products
Pork organ	Boil	Meat and meat products
Pork, no fat	Boil	Meat and meat products
Pork, no fat	Roast	Meat and meat products
Pork, no fat	Stir fry	Meat and meat products
Pork, with fat	Boil	Meat and meat products
Pork, with fat	Roast	Meat and meat products
Pork, with fat	Stir fry	Meat and meat products
Butter, regular	No cooking	Milk and dairy products
Cheese, low fat	No cooking	Milk and dairy products
Cheese, regular	No cooking	Milk and dairy products
Creamer	Reconstitute	Milk and dairy products
Fermented milk	No cooking	Milk and dairy products
Ice cream	No cooking	Milk and dairy products
Infant formulae (0 to 12 mths)	Reconstitute	Milk and dairy products
Margarine	No cooking	Milk and dairy products
Yoghurt, low fat	No cooking	Milk and dairy products
Yoghurt, regular	No cooking	Milk and dairy products
Carrot	Boil	Root and tubers
Carrot	Ready to eat	Root and tubers
Carrot	Stir fry	Root and tubers

French fries	Deep fry	Root and tubers
Lotus root	Soup (liquid)	Root and tubers
Lotus root	Soup (solids)	Root and tubers
Lotus root	Stir fry	Root and tubers
Potato	Bake	Root and tubers
Potato	Boil	Root and tubers
Potato	Deep fry	Root and tubers
Potato	Roast	Root and tubers
Potato	Stir fry	Root and tubers
Pumpkin	Boil	Root and tubers
Sweet potato	Bake	Root and tubers
Sweet potato	Bake	Root and tubers
Sweet potato	Boil	Root and tubers
Sweet potato	Boil	Root and tubers
Sweet potato	Roast	Root and tubers
Sweet potato	Roast	Root and tubers
Sweet potato	Steam	Root and tubers
Yam	Boil	Root and tubers
Yam	Steam	Root and tubers
Fried chips and cracker	No cooking	RTE savouries
Popcorn	No cooking	RTE savouries
Roasted nut snack	No cooking	RTE savouries
Cheese sauce	No cooking	Sauces and condiments
Chilli powder	No cooking	Sauces and condiments
Chilli sauce	No cooking	Sauces and condiments
Chilli sauce	No cooking	Sauces and condiments
Cream sauce	Boil	Sauces and condiments
Fish sauce	No cooking	Sauces and condiments
Mayonnaise	No cooking	Sauces and condiments
Mustard sauce	No cooking	Sauces and condiments
Oyster sauce	No cooking	Sauces and condiments

Pepper, black	No cooking	Sauces and condiments
Pepper, white, powder	No cooking	Sauces and condiments
Sambal chilli/belacan	Stir fry	Sauces and condiments
Satay sauce	Boil	Sauces and condiments
Soy sauce	No cooking	Sauces and condiments
Soy sauce, dark	No cooking	Sauces and condiments
Soy sauce, light	Boil	Sauces and condiments
Soy sauce, light	Ready to eat	Sauces and condiments
Teriyaki sauce	No cooking	Sauces and condiments
Thousand island sauce	No cooking	Sauces and condiments
Tomato sauce	No cooking	Sauces and condiments
Vinegar	No cooking	Sauces and condiments
Wasabi	No cooking	Sauces and condiments
Asparagus	Boil	Stalk, stem and bulb vegetables
Asparagus	Boil	Stalk, stem and bulb vegetables
Bamboo shoot	Boil	Stalk, stem and bulb vegetables
Celery	Boil	Stalk, stem and bulb vegetables
Celery	Stir fry	Stalk, stem and bulb vegetables
Dried lily bulb	Boil	Stalk, stem and bulb vegetables
Garlic	Soup (liquid)	Stalk, stem and bulb vegetables
Garlic	Soup (liquid)	Stalk, stem and bulb vegetables
Garlic	Soup (solids)	Stalk, stem and bulb vegetables
Garlic	Soup (solids)	Stalk, stem and bulb vegetables
Garlic	Stew	Stalk, stem and bulb vegetables
Garlic	Stew	Stalk, stem and bulb vegetables
Garlic	Stir fry	Stalk, stem and bulb vegetables
Garlic	Stir fry	Stalk, stem and bulb vegetables
Ginger	Soup (liquid)	Stalk, stem and bulb vegetables
Ginger	Soup (solids)	Stalk, stem and bulb vegetables
Ginger	Steam	Stalk, stem and bulb vegetables
Ginger	Stir fry	Stalk, stem and bulb vegetables

Lily bulb	Stir fry	Stalk, stem and bulb vegetables
Lily bulb	Stir fry	Stalk, stem and bulb vegetables
Onion	Boil	Stalk, stem and bulb vegetables
Onion	Boil	Stalk, stem and bulb vegetables
Onion	Stir fry	Stalk, stem and bulb vegetables
Onion	Stir fry	Stalk, stem and bulb vegetables
Spring onion	Ready to eat	Stalk, stem and bulb vegetables
Turmeric	Boil	Stalk, stem and bulb vegetables
Tap water	No cooking	Tap water, drinking water
Mock meat	Braised	Vegetable protein
Mock meat (gluten based)	Braised	Vegetable protein
Mock meat (gluten based)	Stir fry	Vegetable protein
Mock meat (soy based)	Braised	Vegetable protein
Mock meat (soy based)	Deep fry	Vegetable protein
Mock meat (soy based)	Stir fry	Vegetable protein

Table S2: Kruskal Wallis test using Chi-Square (df:3) distribution (right tailed) for the four PFAS occurrence means at 99% confidence level (α = 0.01)

Kruskal-Wallis-test parameter	Results
H_0 hypothesis	The occurrence means of all 4 PFAS are assumed to be equal.
P-value	p-value equals 0.530, P(x≤2.2087) = 0.4698.<br>p-value > α, H_0 is accepted.
Test statistic	The test statistic H equals 2.2087, which is in the 99% region of acceptance: [0, 11.3449].
Effect size	Observed effect size η^2 is very small, -0.013. This indicates that the magnitude of the difference between the average is very small.
Multiple comparisons	There is no significant difference between the mean ranks of any pair.

The Kruskal-Wallis H test indicated that there is a non-significant difference in the occurrence means between the different groups, with the magnitude of difference between the average is very small, $\chi^2(3) = 2.21$, p = 0.530, with a mean rank score of 32.2 for PFHxS, 31.93 for PFOA, 29.75 for PFNA, 37.82 for PFOS.

Table S3: One Way ANOVA test for the various cooking method at 95% confidence level ($\alpha = 0.05$) and Tukey HSD

ANOVA for	Anchovy PFOS	Anchovy PFNA	Cockle PFOA	Cockle PFNA
H_0 hypothesis	Mean value for soup solid and pan fry are assumed to be equal		Mean value for uncooked, boil, and stir fry are assumed to be equal	
P-value	0.082 p-value > α, H_0 is accepted	0.198 p-value > α, H_0 is accepted	0.961 p-value > α, H_0 is accepted	0.804 p-value > α, H_0 is accepted
Test statistic	F equals 5.333, in the region of acceptance	F equals 2.375, in the region of acceptance	F equals 0.040, in the region of acceptance	F equals 0.235, in the region of acceptance
Effect size	Effect size f is large (1.15). Large magnitude of the difference averages. η^2 equals 0.57. Group explains 57.1% of the variance from the average	Effect size f is large (0.77). Large magnitude of the difference averages. η^2 equals 0.37. Group explains 37.3% of the variance from the average	Effect size f is small (0.16). Small magnitude of the difference averages. η^2 equals 0.026. Group explains 2.6% of the variance from the average	Effect size f is large (0.4). Large magnitude of the difference averages. η^2 equals 0.14. Group explains 13.5% of the variance from the average
Tukey HSD	There is no significant difference between the means of any pair.	There is no significant difference between the means of any pair.	There is no significant difference between the means of any pair.	There is no significant difference between the means of any pair.
